# Women’s Satisfaction with Gynecological Healthcare Services in a Public Tertiary Facility: A Questionnaire Study

**DOI:** 10.3390/healthcare13243244

**Published:** 2025-12-11

**Authors:** Iwona Gawron, Kamil Derbisz, Dominika Trojnarska, Lucja Zaborowska, Inga Ludwin, Artur Ludwin

**Affiliations:** 1 Chair of Gynecology and Obstetrics, Faculty of Medicine, Jagiellonian University Medical College, Kopernika 23, 31-501 Krakow, Poland; iwona.gawron@uj.edu.pl; 2 Clinical Department of Gynecological Endocrinology and Gynecological Oncology, University Hospital in Krakow, Kopernika 23, 31-501 Krakow, Polanddominika.trojnarska@uj.edu.pl (D.T.); 3 Department of Maternal and Child Health, Institute of Nursing and Midwifery, Faculty of Health Sciences, Jagiellonian University Medical College, Kopernika 25, 31-501 Krakow, Poland; 4 1st Department of Obstetrics and Gynecology, Medical University of Warsaw, Starynkiewicza Sq 1/3, 02-015 Warsaw, Poland; 5 Doctoral School of Medical and Health Sciences, Jagiellonian University Medical College, sw. Lazarza 16, 31-530 Krakow, Poland; 6 Ludwin & Ludwin Private Medical Center, Rakowicka 17/7, 31-510 Krakow, Poland

**Keywords:** patient satisfaction, gynecological healthcare service, organizational culture, public healthcare

## Abstract

**Background:** To evaluate women’s satisfaction with gynecological services at a public referral facility, recognizing the importance of patient satisfaction in assessing healthcare quality. **Methods:** Patient satisfaction was assessed with a 52-item questionnaire, targeting women undergoing laparoscopy/laparotomy, operative and office hysteroscopy, hormonal diagnostics, and others. The impact of patient-specific and hospitalization-related variables on satisfaction indicators and the relationships between provider-specific factors, expectation fulfillment, and readmission willingness were analyzed. Additionally, the institution’s organizational culture was evaluated. **Results:** Analysis of 790 questionnaires revealed a median satisfaction score of 88.48% and scores of 92.31%, 89.42%, 88.18%, 88.33%, and 85% across groups, with the highest satisfaction for laparoscopy/laparotomy (*p* = 0.003). Expectation fulfillment and total satisfaction relied on devoted time and alternatives discussion (both *p* = 0.001 and <0.001, respectively), positively correlating with comprehension, sense of security, and information quality (all *p* < 0.001). Readmission willingness was affected by devoted time (*p* = 0.016) and alternatives discussion (*p* = 0.028), and positively correlated with sense of security (*p* = 0.01). Expectation fulfillment and total satisfaction for office hysteroscopy depended on ongoing information (both *p* < 0.001) and pain aspects, positively correlating with comprehension, communication, and security, but negatively with pain (all *p* < 0.001), without affecting readmission willingness. Significant correlations existed between patient-specific and hospitalization-related variables and satisfaction, expectation fulfillment, and readmission willingness. A hierarchical culture with clan orientation was identified. **Conclusions:** Women’s high satisfaction was primarily linked to information quality. Readmission willingness correlated with a sense of security. Communication was crucial during office hysteroscopy. Public healthcare setting did not affect staff attitudes. Clan-oriented hierarchical culture favored a sense of security.

## 1. Introduction

The transformation of the physician-patient relationship has redefined the physician’s role from merely providing assistance with illness to becoming a healer and quality-of-life enhancer, thereby empowering patients as healthcare consumers whose perspectives increasingly shape quality monitoring and health policy. This change has led to an increased emphasis on patient experience within the healthcare system [1]. Patient satisfaction is not always prioritized in Polish public healthcare, as specialized medical services critical to health prove too costly for individuals or private insurers. Consequently, patients have no alternative but to depend on the public sector, which may negatively impact facility staff’s efforts to deliver a high-quality experience. Furthermore, gynecological care is not considered a priority as the strategic framework for Polish healthcare system development from 2021 to 2027 (available at: https://www.gov.pl/web/zdrowie/zdrowa-przyszlosc-ramy-strategiczne-rozwoju-systemu-ochrony-zdrowia-na-lata-2021-2027-z-perspektywa-do-2030; accessed on 3 October 2025) has concentrated on improving the quality of life for older adults and the mental health of the population. Moreover, healthcare in Poland has been hindered by a relative shortage of resources and a low Gross Domestic Product (GDP) allocation of 6.4% in 2022 compared to other European countries [available from: https://doi.org/10.2908/HLTH_SHA11_HF; accessed on 3 October 2025]. Although preliminary data have indicated an increase to 7.1% of GDP in 2023 and 8.1% in 2024, according to the Central Statistical Office (available at: https://stat.gov.pl/en/topics/health/health/health-care-expenditure-in-20222024,18,5.html; accessed on 23 November 2025), these figures remain lower than the estimated average expenditures of 9.3% in Organisation for Economic Co-operation and Development (OECD) countries (available at: https://www.oecd.org/en/publications/health-at-a-glance-2025_15a55280-en/poland_c3917772-en.html; accessed on 23 November 2025) and 10.0% in European Union countries (available at: https://ec.europa.eu/eurostat/statistics-explained/index.php?title=Healthcare_expenditure_statistics_-_overview; accessed on 23 November 2025). Such an environment promotes systemic inefficiency, characterized by insufficient coordination among healthcare units and disrupted information flow, resulting in prolonged waiting times for scheduled services [2]. While the healthcare sector aims to meet patient needs, a significant deficiency exists in patient awareness of their health and the system’s functioning, with decisions often prioritizing system needs over patient engagement. This is not only due to the inefficient allocation of financial and time resources but also to staff attitudes and the organizational culture of healthcare institutions, which substantially influences employee behavior [3]. As a result of an ongoing transition to a patient-centered approach, driven by a growing awareness that the value of care should be assessed by the individuals receiving it, healthcare systems have begun to incorporate patient-reported measures. The underlying assumption was that a better understanding of patients would improve their experiences with the structure, processes, and outcomes of the healthcare system [4,5]. The Act of 16 June 2023 on the Quality of Care and Patient Safety (available at: https://isap.sejm.gov.pl/isap.nsf/DocDetails.xsp?id=WDU20230001692; accessed on 3 October 2025), has introduced new principles for measuring healthcare quality in Poland. This legislation establishes an internal quality and safety management system aimed at preventing adverse events and ensuring ongoing maintenance and improvement through effectiveness assessments and feedback from patient experience surveys. In anticipation of the forthcoming legislation, a questionnaire was conducted to thoroughly assess patient satisfaction with gynecological services in a public tertiary referral facility. The analysis of patient feedback would provide a framework for identifying trends, patterns, and areas requiring improvement.

## 2. Materials and Methods

A cross-sectional survey prospectively included women hospitalized for gynecological services in both inpatient and day hospital settings for medical or surgical gynecological services from July 2021 to June 2023 at a public tertiary care facility. The study was approved by the Bioethics Committee of Jagiellonian University (no. 1072.6120.127.2021) and conducted in accordance with the Helsinki Declaration, with each participant providing informed written consent. The Research Ethics Committee at the Jagiellonian University Medical College issued a positive opinion regarding the study on the organizational culture of the clinical department (no. 118.0043.1.27.2024).

Women were included in the study consecutively based on their hospitalizations and were recruited on the day of their discharge, provided they gave informed written consent. The distribution of the paper-based questionnaires was conducted by a physician from the research team and was preceded by the provision of information regarding the study’s objectives and detailed instructions for completion. All women completed the questionnaire under similar conditions, and the staff did not interfere with this process. The completed questionnaire forms were submitted anonymously into a designated drop box. Eligible participants were required to be 18 or older, with no exclusion criteria applied. The questionnaire enclosed in Supplement contained 52 questions, 50 of which were closed-ended, enabling respondents to select the answer that most accurately reflected their assessment. The responses were categorized using a multichotomous scale and through ranking. The last two open-ended questions allowed women to voluntarily provide feedback on any positive or negative aspects of the medical care received. Numeric responses were assigned a corresponding point value, while binary yes/no responses were assigned 10/0 points, respectively. Higher scores indicated greater satisfaction. Two questions used a reversed scale: in question 22, a “no” response was awarded 10 points, while a “yes” response was awarded 0 points. In question 35, the scale was reversed, such that a score of 0 was converted to 10 points. The total satisfaction score was presented as a percentage of the possible points that could be attained by each respondent.

The questionnaire began with demographic questions and was structured into sequential sections to be completed according to the type of medical service provided and its subsequent stages, each accompanied by appropriate header information. The services provided were grouped into the following categories: surgery via laparotomy or laparoscopy; hysteroscopy under general anesthesia; hysteroscopy without general anesthesia (office hysteroscopy, OH); hormonal diagnostics for menstrual disorders or infertility; and ‘other’ services, including treatment for early pregnancy complications, procedures related to early pregnancy loss, conservative treatment of gynecological conditions, and procedures for urinary incontinence or pelvic organ prolapse. Assuming approximately 4000 hospitalizations, a minimum sample size of 345 individuals was determined (with a confidence level of 0.95, a consented population proportion of 50%, and a margin of error of 5%), which justified the distribution of 900 questionnaires to achieve an anticipated return rate of less than 50% for fully completed surveys.

An anonymous prospective evaluation of organizational culture was conducted among healthcare personnel using the Organizational Culture Assessment Instrument (OCAI) [6]. The OCAI employs the competing values model, which categorizes organizational culture into four main types: “clan” (collaboration), “adhocracy” (innovation), “hierarchy” (control), and “market” (competition) cultures [6]. Respondents assessed the organization’s current and desired cultures, revealing gaps between the present and preferred cultures.

### 2.1. Statistical Analysis

The analysis of quantitative variables involved calculating descriptive statistics such as mean, standard deviation, median, quartiles, minimum, and maximum values. Qualitative variables were evaluated by computing absolute and percentage frequencies for all possible values. Comparative analyses of qualitative variables across groups utilized the chi-square test (with Yates’ correction for 2 × 2 tables) or Fisher’s exact test when chi-square assumptions were not met. For quantitative variables, the Mann-Whitney U test was employed for two-group comparisons, while the Kruskal-Wallis test was used for three or more groups, followed by Dunnett’s post-hoc test if significant differences were found. Correlations between quantitative variables were assessed using Spearman’s correlation coefficient. A significance level of 0.05 was established, with *p*-values below 0.05 deemed indicative of significant relationships. All analyses were conducted using R software, version 4.5.0 [7].

### 2.2. Questionnaire Validation

The validation included questions with numerical responses as well as binary (yes/no) responses, assigning values of 10 and 0 points, respectively. The scale comprised 33 questions, resulting in a total score ranging from 0 to 330, with higher scores indicating greater satisfaction. Two questions employed a reversed scale: in question 22, a response of “no” was awarded 10 points, while a response of “yes” received 0 points. In question 35, the scale was inverted such that a score of 0 was converted to 10 points, 1 to 9 points, and so forth.

The overall score for the instrument was calculated using data from a sample of 267 women who completed the survey from question 12 to the end, excluding question 38. The average score was 286.13 (SD = 31.14), with scores ranging from 112 to 327, and a median score of 292 (Q1 = 274; Q3 = 306). Confirmatory factor analysis (CFA) was performed confirming one-factor structure by calculating fit indices following modifications suggested by the modification indices [8]. The resulting values were as follows: Standardized Root Mean Residual (SRMR) = 0.066, Comparative Fit Index (CFI) = 0.828, and Root Mean Square Error of Approximation (RMSEA) = 0.059. These values satisfied the criteria for the two-index methodology (SRMR < 0.09, and additionally CFI > 0.96 or RMSEA < 0.06) [8]. The loadings for 31 of the 33 questions were statistically significant (*p* < 0.05) and correlated with the overall score, whereas the loadings for questions 13 and 14 were insignificant, indicating no correlation with the overall score. Subsequently, internal consistency was evaluated by calculating Cronbach’s alpha, yielding a value of 0.804 for the total score, thereby confirming the instrument’s reliability [9]. Furthermore, all items exhibited positive item-total correlations, indicating a favorable alignment with the other items within the scale, which is deemed a highly desirable outcome.

## 3. Results

A total of 900 surveys were distributed, yielding 859 responses (859/900, 95.44%), of which 790 (790/900, 87.78%) were complete, thereby providing a basis for further analysis. The characteristics of the surveyed participants, including demographic data, purpose of hospitalization, health issues, and their responses regarding qualification for the procedure, preoperative period, preparation and conduct of OH, postoperative period, and pre-discharge considerations, were presented in Supplementary Table S1. It was observed that women generally rated the evaluated aspects of healthcare services highly, as the median percentage of the total satisfaction score was 88.48%.

The correlations of age, domicile, education, and profession with the perception of general and mental/emotional health status were depicted in Supplementary Table S2. The general health self-assessment was highest among women aged 31–40 and those with higher education, whereas it was lowest among women over the age of 50 and those with elementary schooling (*p* < 0.001; *p* = 0.001). The mental health self-assessment was highest among women aged 41–50, lowest among those under 20 (*p* < 0.001), and also higher among women in non-health-related professions than among those in health-related professions (*p* = 0.04).

The influence of specific aspects on the perception of treatment with politeness and respect, opportunity to ask questions at discharge, enough time to ask questions, meeting expectations, likelihood of re-hospitalization, negative and positive comments, and total satisfaction score was then assessed and outlined in Table 1.

The rating of being treated with courtesy and respect by staff was highest among women over 50 and lowest among those under 20 (*p* = 0.014), as well as highest among women rating their mental/emotional health as excellent and lowest among those rating it as poor (*p* = 0.014). The percentage of individuals who declared the opportunity to ask questions at discharge was highest among women aged 41–50 and lowest among women under 20 (*p* = 0.004). The percentage of individuals who reported that the physician devoted sufficient time to them was highest among women aged 41–50 and lowest among those aged 21–30 (*p* < 0.001). Women over the age of 50 rated expectation fulfillment significantly higher than women in lower age groups (*p* < 0.001). Women rating their general health as excellent reported higher fulfillment assessments than those rating it lower (*p* < 0.001). Likewise, women assessing their mental/emotional health as excellent, good, or very good reported higher expectation fulfillment than those rating it as average or poor (*p* < 0.001). Women without a history of depression or mood disorders rated expectation fulfillment higher than those with these conditions (*p* = 0.004), and women in relationships rated it significantly higher than single women (*p* = 0.036). Expectation fulfillment was significantly higher among women who waited up to 2 weeks, 2 weeks to 1 month, or 1–3 months for admission compared to those who waited 3–6 months (*p* = 0.011). The percentage of individuals who would choose the same facility for readmission was highest among women rating their general health as excellent and lowest among those rating it as poor (*p* < 0.001). The highest proportion of individuals reporting unaddressed negative aspects of hospitalization was observed among women aged 31 to 40, residents of cities with populations exceeding one million, women with higher education, and those who rated their general health as poor. Conversely, the lowest proportions were noted among individuals under 20, rural residents, those with secondary education, and those rating their health as average (*p* = 0.002, *p* = 0.001, *p* < 0.001, *p* = 0.02, respectively). The proportion identifying unaddressed positive aspects of hospitalization was highest among women aged 21 to 30 and lowest among those over 50 (*p* < 0.001). It was also highest among residents of cities with populations between 100,000 and one million, and lowest among rural residents (*p* = 0.01). Additionally, it was highest among women with higher education and lowest among those with primary education (*p* = 0.002). Total satisfaction scores were significantly higher among women over 50 than those aged 41–50, 31–40, and 21–30 (*p* = 0.012). Scores were also higher among women rating their general health as excellent versus good, average, or poor, and among those rating their health as very good compared to poor (*p* = 0.007). Furthermore, satisfaction was greater among women assessing their mental/emotional health as excellent, very good, or good compared to average (*p* = 0.047). Women without a history of depression or mood disorders reported higher satisfaction than those with such conditions (*p* = 0.022).

The impact of perceived amount of devoted time and the presentation of alternative treatments on expectation fulfillment, likelihood of readmission, and total satisfaction score was depicted in Table 2. Women whose physicians allocated sufficient time and discussed alternative treatments reported significantly higher expectation fulfillment (both *p* = 0.001), willingness to choose the same facility (*p* = 0.016, *p* = 0.028), and total satisfaction score (both *p* < 0.001).

The correlations between subjective assessments of comprehension, sense of security, and quality of information with expectation fulfillment, likelihood of readmission, and total satisfaction score were presented in Table 3. Expectation fulfillment positively correlated with comprehension (r = 0.555, *p* < 0.001), sense of security (r = 0.382, *p* < 0.001), and information quality (r = 0.562, *p* < 0.001). Univariable logistic regression showed that each additional point in sense of security increased the odds of choosing the same facility for readmission by 2.25 (OR = 2.25, *p* = 0.01). Higher ratings of comprehension, sense of safety, and quality of information were associated with increased total satisfaction scores (all *p* < 0.001).

The impact of selected variables on expectation fulfillment and willingness to repeat office hysteroscopy (OH) among women was illustrated in Table 4. Expectation fulfillment was significantly higher for those whose physicians allocated sufficient time (*p* = 0.001), presented alternative treatments (*p* < 0.001), informed about forthcoming actions (*p* < 0.001), and warned of potential discomfort (*p* = 0.001).

The network of significant relationships between patient-dependent and provider-dependent factors concerning selected aspects of patient experience was illustrated in Supplementary Figure S1.

Additionally, women who did not report pain had higher expectation fulfillment than those whose procedures were not interrupted due to pain (*p* = 0.04). None of the variables affected the willingness to be readmitted to the facility for a repeat hysteroscopy if necessary. Total satisfaction scores were significantly higher among women who felt their physician allocated sufficient time, presented alternatives, provided ongoing information, and warned about potential discomfort (all *p* < 0.001), compared to those who reported the opposite.

The correlations among comprehension, sense of security, quality of information, intensity of pain, and quality of communication during hysteroscopy with meeting expectations, likelihood of re-admission, and total satisfaction score were shown in Table 5. Meeting expectations and total satisfaction score positively correlated (*p* < 0.05, r > 0) with all parameters, except for pain, which exhibited a significant negative correlation (*p* = 0.001, r = −0.21). In contrast, the likelihood of re-admission significantly correlated only with comprehension (OR = 1.672, *p* = 0.04) and quality of communication (OR = 1.355, *p* = 0.047) in univariable logistic regression models.

Expectation fulfillment was significantly higher among women who received ‘other’ interventions, underwent OH, and had laparotomy or laparoscopy, as well as those who had hysteroscopy under general anesthesia, compared to those who underwent hormonal diagnostics (*p* < 0.001). Willingness to be readmitted if needed again did not differ between groups (*p* = 0.967). Total satisfaction scores were higher for women who underwent laparotomy or laparoscopy compared to those who had OH or ‘other’ services, and for those undergoing hormonal diagnostics compared to those receiving ‘other’ services (*p* = 0.003) (Table 6).

Sixty employees completed the OCAI questionnaire, with the models presented in Supplementary Figure S2. The current culture was oriented towards a traditional management approach, characterized by formal structures and control, as indicated by a high score of 35.01% for “hierarchy.” Employees expressed a desire for a more community-oriented atmosphere emphasizing collaboration and decision-making involvement, reflected in the increase of the “clan” percentage from 21.22% to 29.84% in the desired culture. There was a noticeable need to reduce the focus on competition and performance, signaling a preference for a more balanced management approach, as evidenced by the decrease in the “market” percentage from 26.86% to 17.13% and the increase in the “adhocracy” percentage from 16.91% to 23.35%.

## 4. Discussion

In recent years, healthcare institutions have increasingly recognized the importance of transparency in patient satisfaction outcomes to identify improvement areas and enhance patient engagement. In some systems, patient satisfaction surveys are legally mandated, with findings playing a crucial role in institutional evaluations and influencing funding allocation by public payers.

### 4.1. Comparison of Results with Benchmarks for the Public Sector

Analysis of the obtained results indicates that women reported an overall satisfaction with the gynecological services received from the public healthcare system. Since there is no universal benchmark for overall patient satisfaction, as various systems and institutions employ different measurement methods, such as the Likert scale [10], the Net Promoter Score [11], the American Hospital Consumer Assessment of Healthcare Providers and Systems [12], or commercial surveys like Press Ganey [13], the comparison of the outcomes to international reference points was not feasible. In Poland, patient satisfaction data are continuously collected by the Health Quality Monitoring Centre, and the results it provides can be considered approximate national indicators. Data collected by this Centre between 2022 and 2023 from over 90,000 anonymous patient surveys conducted across more than 170 hospitals (published in a press release at: https://www.cmj.org.pl/czytaj/37; accessed on 23 November 2025) indicated a favorable level of patient satisfaction. Specifically, 86% of respondents were satisfied with medical care, 89% positively evaluated communication from staff, 90% felt safe, 80% reported health improvements post-hospitalization, 75% had no complaints about conditions, and 84% would recommend the facility. The performance of the examined facility was consistent with these generalized results, featuring a total satisfaction score of 88.48%, with 88.4% of respondents declaring a sense of security, 78.73% of patients not expressing negative comments, and 93.70% indicating a willingness to return for future services. To illustrate the level of patient satisfaction with the healthcare system in Poland compared to other countries, reference can be made to the Health at a Glance 2025 report (available at: https://www.oecd.org/en/publications/health-at-a-glance-2025_15a55280-en/poland_c3917772-en.html; accessed on 23 November 2025), which indicated that 51% of Poles expressed satisfaction with the availability of high-quality healthcare, whereas the average satisfaction rate among countries in the Organisation for Economic Co-operation and Development (OECD) was 64%. Considering Poland’s 32nd position out of 35 countries in the final edition of the European Health Consumer Index (EHCI) from 2018, which evaluated European healthcare systems based on waiting times, treatment outcomes, and service quality, it can be inferred that patient satisfaction remains consistently suboptimal (available at: https://santesecu.public.lu/dam-assets/fr/publications/e/euro-health-consumer-index-2018/euro-health-consumer-index-2018.pdf; accessed on 23 November 2025).

The interpretation of research findings on patient experiences in healthcare is complicated not only by political factors but also by a range of influencing elements, including patient-dependent factors such as health self-awareness and immutable demographic characteristics, along with provider-related aspects such as communication and management [14].

### 4.2. Patient-Dependent Factors Influencing Satisfaction with Gynecological Healthcare Services

The variable of self-assessed health status is influenced by many factors, with poorer assessments being more often reported by older individuals, residents of rural areas, and those with lower education, income, or employment status [15,16,17]. Our findings aligned with previous research, particularly regarding age and education, showing that general health assessments were lowest among women over 50 and those with elementary education, while mental/emotional health self-assessments were lowest among women under 20. While women in health-related professions exhibited significantly worse mental/emotional health than those in non-medical fields—consistent with evidence of a mental health crisis among healthcare workers [18,19]—this disparity did not vary by education level, contradicting studies from other countries [20,21]. The better the participants rated their general and mental/emotional health, the more their expectations regarding healthcare were met, leading to a higher total satisfaction score. Conversely, those who rated their general health poorly were more likely to provide negative feedback and less inclined to return to the same facility. Age has been recognized as a key demographic factor influencing healthcare quality assessments [22], correlating with the highest number of examined aspects of medical services in this study. Older women reported feeling treated with greater kindness and respect, experienced more opportunities to ask questions, and expressed higher satisfaction with their time spent with physicians, leading to a greater expectation fulfillment and total satisfaction score compared to younger patients. In prior research, young adults preferred seeking healthcare to relying on self-care for minor illnesses [23]. Healthcare providers viewed this demographic as overly dependent, expecting immediate recovery and full access to tests and treatments, while showing less willingness to manage symptoms independently [24]. The results of this study aligned with previous findings, showing that younger generations had higher expectations of healthcare providers, while older individuals were more accustomed to interactions with the public healthcare system. Among healthcare organizational aspects, shorter waiting times were associated with greater expectation fulfillment, as anticipated [25], but not with a higher total satisfaction score.

### 4.3. Provider-Dependent Factors Influencing Patient Experience with Gynecological Healthcare Services

The literature has indicated that sense of safety, trust in medical staff, and perceptions of being heard and respected are key contributors to positive patient experiences and are central to patient-centered care [26,27,28]. Previous studies have demonstrated that individualized care, staff kindness, treatment outcomes, and discharge organization [29] are the most significant predictors of patients’ likelihood to return and overall satisfaction, although these outcomes should not be regarded as synonymous [30]. Consistent with other studies [14,31], expectation fulfillment and total satisfaction score were significantly and positively associated with several provider-dependent factors, including adequate time spent, discussions of alternative treatment options, communication quality—including clarity and comprehensiveness of information—and a sense of security. Interestingly, the opportunity to ask questions (question 13) and the receipt of satisfactory answers (question 14) did not significantly correlate with overall satisfaction with the provided services. In this study, the common factors influencing the willingness to return across most service types included allotted time, discussion of alternatives, and a sense of security, but not information transfer and communication factors. This highlights the significance of non-verbal determinants of security perception in the healthcare provider-patient relationship, most likely the degree of attention that patients perceive as received, rather than merely the act of transmitting information, alongside intangible factors shaped by the organization’s culture [32]. Conversely, participants undergoing invasive procedures with patient awareness, such as OH, emphasized the importance of comprehension, a sense of security, and high-quality ongoing communication, with the latter significantly influencing their willingness to be readmitted. Furthermore, effective pain management was acknowledged as a crucial element for enhancing the overall service experience; however, unlike communication, it did not influence the decision to reselect the same service provider in the future. It has been previously noted that meeting patients’ expectations regarding the amount and type of needed information poses a challenge for healthcare providers [33], a notion that our study has supported. It can therefore be concluded that improving the process of conscious information provision, as well as the art of communication—in the case of invasive procedures conducted with patient awareness—will contribute to enhancing the patient experience in healthcare.

### 4.4. Organizational Culture-Related Factors Influencing Patient Experience in Gynecological Healthcare Services

Achieving patient-centered care and effective communication is complex due to various barriers, including institutional, communicative, environmental, and individual factors [27]. Healthcare professionals must identify these barriers and facilitators, recognizing their interconnected influence on clinical encounters. On one hand, the traditional hierarchical management model identified in the studied facility, characterized by structured processes, may have reinforced patients’ sense of security, positively impacting study outcomes. Contrary to assumptions, staff awareness of patients’ reliance on public healthcare did not negatively impact staff attitudes, nor did it lead to a compromised patient experience. On the other hand, it should be recognized that employee perspectives reflecting the need for individual consideration in management and treatment likely contributed to positive outcomes. Staff expectations regarding future changes in organizational culture have been reflected in previous research, indicating that adopting a clan culture can enhance care coordination in hospitals [34]. Consequently, it can be inferred that transitioning to a clan culture would elevate the patient experience in healthcare.

### 4.5. Service Type-Dependent Factors Influencing Patient Satisfaction with Gynecological Healthcare Services

The available literature has linked conservative therapy, shorter hospital stays, and perceived improvement following treatment to patient satisfaction [35], a trend also supported by the obtained results. Expectation fulfillment was highest for conservative treatments for gynecological conditions and procedures addressing pelvic organ prolapse or urinary incontinence aimed at improving quality of life, followed by other gynecological surgeries. However, this did not correspond with the total satisfaction score, which was highest for laparoscopy/laparotomy procedures and lowest for ‘other’ procedures. The lowest fulfillment rate was recorded for purely diagnostic hospitalizations related to ovulation disorders and infertility. In the evaluated department, women with suspected endocrinological disorders undergo multiple laboratory tests requiring repeated blood draws, with some results delayed until days after discharge, hindering final diagnosis and treatment. Conversely, surgical procedures typically yield more immediate outcomes, potentially enhancing patient satisfaction. Interestingly, variations in satisfaction with specific medical services did not correlate with the likelihood of patient return.

### 4.6. Practical Implications of the Study’s Findings for Enhancing the Patient Experience with Gynecological Healthcare Services

The results of the study indicate several policy-relevant strategies for enhancing patient-centered gynecological care within public facilities. Training healthcare staff in empathetic communication and shared decision-making may strengthen both the informational and relational dimensions of care, which, according to the study, are closely associated with an increase in patient satisfaction [36,37]. Incorporating systematic, patient-driven feedback into service redesign could further align gynecology units with patients’ needs and expectations [38]. Moreover, fostering interprofessional teamwork consistent with clan-oriented organizational cultures may enhance patients’ sense of security and contribute to more cohesive and responsive care delivery [39]. At the pre-admission stage, it is essential to consider the implementation of online communication measures [40] involving gynecologists and anesthesiologists, particularly with regard to informing patients about preparations for gynecological procedures and anesthetic aspects. The literature has indicated that engaging physicians from various specialties significantly enhances patient adherence to medical recommendations by increasing the perceived comprehensiveness and credibility of the advice [40], potentially leading to better hospitalization outcomes and greater patient satisfaction.

### 4.7. Limitations and Strengths

Among the limitations of this study are its single-center design and its confinement to the public health sector. While participants rated healthcare services positively, this assessment was conducted at discharge and did not account for potential distant complications, which should be considered when interpreting the results [33,41]. Another limitation of the study was the predominance of women who underwent hormonal diagnostics and OH, which was reflective of the center’s profile. The impact of immutable factors related to healthcare providers, such as age, gender, ethnicity, and marital status, known to affect patient evaluations, was not investigated [42]. However, a notable strength was the remarkably high survey response rate, which significantly exceeded the minimum requirements for sample size. This reflects the strong motivation of both parties involved in the study—the healthcare provider and the recipient—to collaborate in the pursuit of enhancing the healthcare experience.

## 5. Conclusions

Women positively assessed the investigated aspects of gynecological services. The most significant demographic factor affecting this evaluation was age. Satisfaction with the healthcare services correlated with the quality of information received and the likelihood of readmission with a sense of security. The quality of effective communication was particularly important during OH conducted without general anesthesia. The strong connections among satisfaction, information quality, and perceived security underscore the urgent need for policies that establish standardized clear communication and enhance patient-centered interactions. Patients’ reliance on public healthcare did not adversely affect staff attitudes. Furthermore, a hierarchical management model that encouraged a shift toward a clan culture fostered patients’ sense of security. Therefore, transitioning from hierarchical to clan-oriented management structures is imperative for further enhancing patients’ sense of security and should be a priority in organizational policy.

## Figures and Tables

**Table 1:** The relationships of specific aspects with the perception of treatment with politeness and respect, opportunity to ask questions at discharge, sufficient time to ask questions, fulfillment of expectations, likelihood of re-hospitalization, negative comments, and positive comments.

Parameter	Group	Treatment with Politeness and Respect				Opportunity to Ask Questions at Discharge			Enough Time to Ask Questions			Meeting Expectations								Likelihood of Re-Hospitalization			Negative Comments			Positive Comments			Total Satisfaction Score [%]							
		Yes	Rather Yes	No	*p*	Yes	No	*p*	Yes	No	*p*	Mean	SD	Median	Min	Max	Q1	Q3	*p* ^b^	Yes	No	*p*	Yes	No	*p*	Yes	No	*p*	Mean	SD	Median	Min	Max	Q1	Q3	*p*
Age	<20 years-A (N = 47)	34 (72.34%)	13 (27.66%)	0 (0.00%)	*p* = 0.014 *	42 (89.36%)	5 (10.64%)	*p* = 0.004 *	40 (85.11%)	7 (14.89%)	*p* < 0.001 *	7.66	1.58	8.0	4	10	7	9.00	*p* < 0.001 * E > C, B, A D, C > B, A	47 (100.00%)	0 (0.00%)	*p* = 0.398	5 (10.64%)	42 (89.36%)	*p* = 0.002 *	6 (12.77%)	41 (87.23%)	*p* < 0.001 *	85.23	14.30	88.33	35.00	100	78.33	96.67	*p* ^c^ = 0.012 * E > D, B, C
	21–30 years-B (N = 241)	181 (75.1%)	59 (24.48%)	1 (0.41%)		228 (94.61%)	13 (5.39%)		197 (81.74%)	44 (18.26%)		7.82	1.80	8.0	1	10	7	9.00		235 (97.51%)	6 (2.49%)		46 (19.09%)	195 (80.91%)		66 (27.39%)	175 (72.61%)		82.27	18.39	88.33	18.46	100	75.45	96.67	
	31–40 years-C (N = 274)	233 (85.04%)	41 (14.96%)	0 (0.00%)		266 (97.08%)	8 (2.92%)		257 (93.80%)	17 (6.20%)		8.47	1.57	9.0	1	10	8	10.00		269 (98.18%)	5 (1.82%)		76 (27.74%)	198 (72.26%)		61 (22.26%)	213 (77.74%)		83.82	16.76	87.88	9.62	100	80.38	94.24	
	41–50 years-D (N = 121)	103 (85.12%)	17 (14.05%)	1 (0.83%)		120 (99.17%)	1 (0.83%)		115 (95.04%)	6 (4.96%)		8.70	1.58	9.0	3	10	8	10.00		117 (96.69%)	4 (3.31%)		29 (23.97%)	92 (76.03%)		15 (12.40%)	106 (87.60%)		86.33	12.68	89.09	22.69	100	80.61	95.38	
	>50 years-E (N = 107)	95 (88.78%)	12 (11.21%)	0 (0.00%)		106 (99.07%)	1 (0.93%)		99 (92.52%)	8 (7.48%)		8.97	1.72	10.0	0	10	8	10.00		106 (99.07%)	1 (0.93%)		12 (11.21%)	95 (88.79%)		10 (9.35%)	97 (90.65%)		89.70	9.59	91.92	56.54	100	84.92	96.15	
Domicile	Village (N = 252)	210 (83.33%)	41 (16.27%)	1 (0.40%)	*p* = 0.384	248 (98.41%)	4 (1.49%)	*p* = 0.065	232 (92.06%)	20 (7.94%)	*p* = 0.131	8.44	1.61	9.0	0	10	8	10.00	*p* = 0.094	249 (98.81%)	3 (1.19%)	*p* = 0.164	34 (13.49%)	218 (86.51%)	*p* = 0.001 *	35 (13.89%)	217 (86.11%)	*p* = 0.01 *	86.16	15.82	91.21	18.46	100	82.42	96.67	*p* ^d^ = 0.077
	City < 50,000 (N = 96)	76 (79.17%)	19 (19.79%)	1 (1.04%)		92 (95.83%)	4 (4.16%)		85 (88.54%)	11 (11.46%)		8.43	1.85	9.0	1	10	8	10.00		93 (96.88%)	3 (3.12%)		16 (16.67%)	80 (83.33%)		15 (15.62%)	81 (84.38%)		84.49	15.24	88.46	23.08	100	78.76	95.19	
	City 50,000–100,000 (N = 68)	53 (77.94%)	14 (20.59%)	1 (1.47%)		65 (95.59%)	3 (4.41%)		62 (91.18%)	6 (8.82%)		8.54	1.62	9.0	3	10	8	10.00		67 (98.53%)	1 (1.47%)		14 (20.59%)	54 (79.41%)		15 (22.06%)	53 (77.94%)		84.86	14.78	88.56	23.08	100	78.33	94.09	
	City 100,000–1,000,000 (N = 220)	177 (80.45%)	42 (19.09%)	1 (0.45%)		208 (94.55%)	12 (5.45%)		188 (85.45%)	32 (14.55%)		8.08	1.79	8.0	1	10	7	10.00		212 (96.36%)	8 (3.63%)		60 (27.27%)	160 (73.73%)		59 (26.82%)	161 (73.18%)		82.43	17.44	87.44	9.62	100	78.16	95.00	
	City > 1,000,000 (N = 154)	128 (83.12%)	26 (16.88%)	0 (0.00%)		149 (96.75%)	5 (3.25%)		141 (91.56%)	13 (8.44%)		8.34	1.70	8.5	1	10	8	10.00		153 (99.35%)	1 (0.65%)		44 (28.57%)	110 (71.43%)		34 (22.08%)	120 (77.92%)		85.16	14.74	88.41	15.38	100	80.00	95.69	
Education	Elementary (N = 26)	20 (76.92%)	6 (23.08%)	0 (0.00%)	*p* = 0.071	24 (92.31%)	2 (7.69%)	*p* = 0.285	23 (88.46%)	3 (11.54%)	*p* = 0.845	7.81	1.50	8.0	5	10	7	8.75	*p* = 0.187	26 (100.00%)	0 (0.00%)	*p* = 0.325	3 (11.54%)	23 (88.46%)	*p* < 0.001 *	3 (11.54%)	23 (88.46%)	*p* = 0.002 *	86.43	11.38	87.50	58.33	100	81.25	96.67	*p* ^d^ = 0.236
	Middle (N = 193)	145 (75.13%)	48 (24.87%)	0 (0.00%)		187 (96.89%)	6 (3.11%)		171 (88.60%)	22 (11.40%)		8.42	1.67	9.0	0	10	8	10.00		192 (99.48%)	1 (0.52%)		16 (8.29%)	177 (91.71%)		23 (11.92%)	170 (88.08%)		85.92	14.42	90.00	22.69	100	78.33	96.67	
	Technical (N = 44)	35 (79.55%)	8 (18.18%)	1 (2.27%)		43 (97.73%)	1 (2.27%)		38 (86.36%)	6 (13.64%)		8.21	2.05	9.0	3	10	7	10.00		42 (95.45%)	2 (4.55%)		6 (13.64%)	38 (86.36%)		6 (13.64%)	38 (86.36%)		86.81	14.04	92.21	46.67	100	82.69	96.74	
	Higher (N = 527)	445 (84.44%)	80 (15.18%)	2 (0.38%)		508 (96.39%)	19 (3.61%)		476 (90.32%)	51 (9.68%)		8.33	1.71	9.0	1	10	8	10.00		514 (97.53%)	13 (2.47%)		144 (27.32%)	383 (72.68%)		126 (23.91%)	401 (76.09%)		83.86	16.80	88.33	9.62	100	79.66	95.00	
Health-related profession	Yes (N = 145)	116 (80.00%)	28 (19.31%)	1 (0.69%)	*p* = 0.349	137 (94.48%)	8 (5.52%)	*p* = 0.156	125 (86.21%)	20 (13.79%)	*p* = 0.13	8.32	1.98	9.0	1	10	8	10.00	*p* = 0.354	143 (98.62%)	2 (1.38%)	*p* = 1	27 (18.62%)	118 (81.38%)	*p* = 0.444	35 (24.14%)	110 (75.86%)	*p* = 0.197	83.46	17.71	88.46	18.46	100	78.33	96.15	*p* ^e^ = 0.797
	No (N = 645)	528 (81.86%)	114 (17.67%)	3 (0.47%)		625 (96.90%)	20 (3.11%)		583 (90.39%)	62 (9.61%)		8.33	1.65	9.0	0	10	7	10.00		631 (97.83%)	14 (2.17%)		141 (21.86%)	504 (78.14%)		122 (18.91%)	523 (81.09%)		84.87	15.54	88.48	9.62	100	79.70	95.77	
General health	Excellent-A (N = 24)	21 (87.50%)	3 (12.50%)	0 (0.00%)	*p* = 0.091	24 (100.00%)	0 (0.00%)	*p* = 0.5	23 (95.83%)	1 (4.17%)	*p* = 0.057	9.25	0.90	9.5	7	10	9	10.00	*p* < 0.001 * A > B, C, D, E B > D, E C > E	24 (100.00%)	0 (0.00%)	*p* < 0.001 *	7 (29.17%)	17 (70.83%)	*p* = 0.02 *	5 (20.83%)	19 (79.17%)	*p* = 0.359	90.68	11.82	93.94	45.00	100	88.39	97.35	*p* ^c^ = 0.007 * A > C, D, E B > E
	Very good-B (N = 261)	221 (84.67%)	40 (15.33%)	0 (0.00%)		256 (98.08%)	5 (1.92%)		242 (92.72%)	19 (7.28%)		8.51	1.57	9.0	1	10	8	10.00		255 (97.70%)	6 (2.30%)		71 (27.20%)	190 (72.80%)		56 (21.46%)	205 (78.54%)		86.09	15.02	90.00	9.62	100	80.91	96.54	
	Good-C (N = 402)	324 (80.60%)	76 (18.91%)	2 (0.50%)		382 (95.02%)	20 (4.98%)		352 (87.56%)	50 (12.44%)		8.33	1.62	9.0	0	10	7	10.00		399 (99.25%)	3 (0.75%)		71 (17.66%)	331 (82.34%)		74 (18.41%)	328 (81.59%)		84.09	16.12	88.18	15.38	100	78.94	95.68	
	Average-D (N = 94)	71 (75.53%)	21 (22.34%)	2 (2.13%)		91 (96.81%)	3 (3.19%)		85 (90.43%)	9 (9.57%)		7.80	2.20	8.0	1	10	7	10.00		89 (94.68%)	5 (5.32%)		16 (17.02%)	78 (82.98%)		18 (19.15%)	76 (80.85%)		82.34	17.40	86.67	18.46	100	77.31	94.96	
	Poor-E (N = 9)	7 (77.78%)	2 (22.22%)	0 (0.00%)		9 (100.00%)	0 (0.00%)		6 (66.67%)	3 (33.33%)		6.22	2.73	5.0	2	10	5	8.00		7 (77.78%)	2 (22.22%)		3 (33.33%)	6 (66.67%)		4 (44.44%)	5 (55.56%)		72.52	20.65	73.33	37.69	100	58.33	89.62	
Mental/Emotional Health	Excellent-A (N = 56)	51 (91.07%)	5 (8.93%)	0 (0.00%)	*p* = 0.014 *	54 (96.43%)	2 (3.57%)	*p* = 0.659	54 (96.43%)	2 (3.57%)	*p* = 0.091	8.77	1.66	9.0	2	10	8	10.00	*p* < 0.001 * A, C, B > D, E	53 (94.64%)	3 (5.36%)	*p* = 0.124	13 (23.21%)	43 (76.79%)	*p* = 0.951	11 (19.64%)	45 (80.36%)	*p* = 0.581	87.21	13.99	90.38	22.69	100	83.33	95.15	*p* ^c^ = 0.047 * A, B, C > D
	Very good-B (N = 275)	224 (81.45%)	50 (18.18%)	1 (0.36%)		266 (96.73%)	9 (3.27%)		248 (90.18%)	27 (9.82%)		8.40	1.71	9.0	1	10	8	10.00		270 (98.18%)	5 (1.82%)		57 (20.73%)	218 (79.27%)		49 (17.82%)	226 (82.18%)		85.16	15.72	90.00	15.38	100	80.00	96.15	
	Good-C (N = 315)	261 (82.86%)	53 (16.83%)	1 (0.32%)		305 (96.83%)	10 (3.17%)		282 (89.52%)	33 (10.48%)		8.42	1.70	9.0	0	10	8	10.00		311 (98.73%)	4 (1.27%)		68 (21.59%)	247 (78.41%)		62 (19.68%)	253 (80.32%)		84.89	16.19	88.79	9.62	100	80.00	95.77	
	Average-D (N = 128)	96 (75.00%)	31 (24.22%)	1 (0.78%)		122 (95.31%)	6 (4.68%)		112 (87.50%)	16 (12.50%)		7.88	1.60	8.0	3	10	7	9.00		124 (96.88%)	4 (3.12%)		26 (20.31%)	102 (79.69%)		32 (25.00%)	96 (75.00%)		81.92	16.32	86.60	18.46	100	77.74	92.35	
	Poor-E (N = 15)	11 (73.33%)	3 (20.00%)	1 (6.67%)		14 (93.33%)	1 (6.67%)		11 (73.33%)	4 (26.67%)		7.27	2.25	7.0	1	10	7	8.00		15 (100.00%)	0 (0.00%)		4 (26.67%)	11 (73.33%)		3 (20.00%)	12 (80.00%)		81.54	18.40	86.67	35.00	100	71.67	96.67	
Mood disorders/depression	Yes (N = 133)	105 (78.95%)	27 (20.30%)	1 (0.75%)	*p* = 0.231	128 (96.24%)	5 (3.76%)	*p* = 0.777	117 (87.97%)	16 (12.03%)	*p* = 0.496	8.01	1.69	8.0	3	10	7	9.00	*p* = 0.004 *	129 (96.99%)	4 (3.01%)	*p* = 0.272	31 (23.31%)	102 (76.69%)	*p* = 0.618	24 (18.05%)	109 (81.95%)	*p* = 0.634	82.68	15.64	86.67	22.69	100	76.67	94.55	*p* ^e^ = 0.022 *
	No (N = 657)	539 (82.04%)	115 (17.50%)	3 (0.45%)		634 (96.50%)	23 (3.6%)		591 (89.95%)	66 (10.05%)		8.39	1.71	9.0	0	10	8	10.00		645 (98.17%)	12 (1.83%)		137 (20.85%)	520 (79.15%)		133 (20.24%)	524 (79.76%)		85.00	16.01	89.39	9.62	100	80.00	96.15	
Relationship Status	Single (N = 163)	130 (79.75%)	33 (20.25%)	0 (0.00%)	*p* = 0.638	157 (96.32%)	6 (3.68%)	*p* = 0.429	140 (85.89%)	23 (14.11%)	*p* = 0.072	8.12	1.72	8.0	1	10	7	10.00	*p* = 0.036 *	160 (98.16%)	3 (1.84%)	*p* = 1	31 (19.02%)	132 (80.98%)	*p* = 0.485	36 (22.09%)	127 (77.91%)	*p* = 0.505	85.55	14.66	88.33	15.38	100	78.33	96.67	*p* ^e^ = 0.344
	In a relationship (N = 627)	514 (81.98%)	109 (17.38%)	4 (0.64%)		605 (96.49%)	22 (3.51%)		568 (90.59%)	59 (9.41%)		8.38	1.71	9.0	0	10	8	10.00		614 (97.93%)	13 (2.07%)		137 (21.85%)	490 (78.15%)		121 (19.30%)	506 (80.7%)		84.37	16.28	88.79	9.62	100	80.00	95.00	
Waiting time for the admission	<2 weeks-A (N = 184)	149 (80.98%)	34 (18.48%)	1 (0.54%)	*p* = 0.353	181 (98.37%)	3 (1.63%)	*p* = 0.558	170 (92.39%)	14 (7.61%)	*p* = 0.254	8.55	1.54	9.0	1	10	8	10.00	*p* = 0.011 * A, C, B > D	182 (98.91%)	2 (1.09%)	*p* = 0.549	45 (24.46%)	139 (75.54%)	*p* = 0.319	33 (17.93%)	151 (82.07%)	*p* = 0.352	80.61	22.96	88.40	9.62	100	77.15	96.67	*p* ^d^ = 0.349
	2 weeks—1 month-B (N = 124)	102 (82.26%)	22 (17.74%)	0 (0.00%)		122 (98.39%)	2 (1.61%)		107 (86.29%)	17 (13.71%)		8.42	1.61	9.0	1	10	7	10.00		122 (98.39%)	2 (1.61%)		25 (20.16%)	99 (79.84%)		24 (19.35%)	100 (80.65%)		86.53	11.19	88.86	45.00	100	78.33	95.19	
	1–3 months-C (N = 219)	189 (86.30%)	30 (13.70%)	0 (0.00%)		210 (95.89%)	9 (4.11%)		202 (92.24%)	17 (7.76%)		8.47	1.66	9.0	1	10	8	10.00		215 (98.17%)	4 (1.83%)		52 (23.74%)	167 (76.26%)		39 (17.81%)	180 (82.19%)		86.83	12.68	90.00	15.38	100	82.12	95.45	
	3–6 months-D (N = 193)	148 (76.68%)	42 (21.76%)	3 (1.56%)		184 (95.34%)	9 (4.66%)		168 (87.05%)	25 (12.95%)		7.91	1.98	8.0	0	10	7	9.00		185 (95.85%)	8 (4.15%)		36 (18.65%)	157 (81.35%)		48 (24.87%)	14 (75.13%)		85.09	13.80	88.33	33.94	100	79.23	95.76	
	>6 months-E (N = 70)	56 (80.00%)	14 (20.00%)	0 (0.00%)		65 (92.86%)	5 (7.14%)		61 (87.14%)	9 (12.86%)		8.27	1.56	8.0	5	10	7	10.00		70 (100.00%)	0 (0.00%)		10 (14.29%)	60 (85.71%)		13 (18.57%)	57 (81.43%)		83.47	13.65	84.81	35.00	100	77.21	92.65	

*p*—chi-squared or Fisher’s exact test, *p* ^b^—2 groups comparison: Mann-Whitney test; >2 groups comparison: Kruskal-Wallis test + post-hoc analysis (Dunn test), *p* ^c^—Kruskal-Wallis test + post-hoc analysis (Dunn test), *p* ^d^—Kruskal-Wallis test, *p* ^e^—Mann-Whitney test, SD—standard deviation, Q1—lower quartile, Q3—upper quartile, and * statistically significant (*p* < 0.05).

**Table 2:** The relationships between the subjective assessment of the amount of time dedicated and the presentation of alternative treatment methods, and the fulfillment of expectations and the likelihood of readmission.

Parameter	Group	Meeting Expectations								Likelihood of Re-Hospitalization				Total Satisfaction Score [%]							
		Mean	SD	Median	Min.	Max.	Q1	Q3	*p* ^a^	Yes	No	Unknown	*p* ^b^	Mean	SD	Median	Min.	Max.	Q1	Q3	*p* ^a^
Allotting enough time	Yes (N = 197)	8.79	1.46	9	0	10	8.00	10.0	*p* = 0.001 *	194 (98.48%)	1 (0.51%)	2 (1.02%)	*p* = 0.016 *	89.06	10.64	92.31	45.00	100.00	84.23	96.15	*p* ^a^ < 0.001 *
	No (N = 16)	7.06	2.11	7	4	10	5.75	8.5		14 (87.50%)	2 (12.50%)	0 (0.00%)		68.46	15.05	70.00	37.69	90.77	63.08	76.35	
Alternatives	Yes (N = 148)	8.89	1.49	9	0	10	8.00	10.0	*p* = 0.001 *	146 (98.65%)	0 (0.00%)	2 (1.35%)	*p* = 0.028 *	91.31	9.18	94.23	51.15	100.00	88.46	98.08	*p* < 0.001 *
	No (N = 65)	8.14	1.68	8	3	10	7.00	10.0		62 (95.38%)	3 (4.62%)	0 (0.00%)		78.86	13.97	82.69	37.69	96.15	71.92	87.31	

*p* ^a^—Mann-Whitney test, *p* ^b^—chi-squared or Fisher’s exact test, SD—standard deviation, Q1—lower quartile, Q3—upper quartile, and * statistically significant (*p* < 0.05).

**Table 3:** The correlations between the subjective assessment of understanding, sense of safety, and quality of information provided, and the fulfillment of expectations and the likelihood of readmission.

Parameter	Meeting Expectations		Likelihood of Re-Hospitalization				Total Satisfaction Score [%]	
	Spearman’s Correlation Coefficient	*p*	OR	95%CI		*p* ^a^	Spearman’s Correlation Coefficient	*p*
Comprehension	r = 0.555	<0.001 *	1.718	0.94	3.138	0.078	r = 0.55	*p* < 0.001 *
Sense od security	r = 0.382	<0.001 *	2.25	1.214	4.171	0.01 *	r = 0.459	*p* < 0.001 *
Quality of information	r = 0.562	<0.001 *	1.421	0.999	2.022	0.051	r = 0.608	*p* < 0.001 *

*p* ^a^—univariate logistic regression, OR—odds ratio, 95%CI—95% confidence interval, and * statistically significant (*p* < 0.05).

**Table 4:** The comparison of expectation fulfillment and willingness to undergo repeat procedures among women who underwent office hysteroscopy, based on selected variables.

Parameter	Group	Meeting Expectations *p*								Likelihood of Re-Admission			Total Satisfaction Score [%]							
		Mean	SD	Median	Min.	Max.	Q1	Q3	*p* ^a^	Yes	No	*p* ^b^	Mean	SD	Median	Min.	Max.	Q1	Q3	*p* ^a^
Allotting enough time	Yes (N = 244)	8.76	1.37	9	3	10	8.0	10.00	*p* = 0.001 *	241 (98.77%)	3 (1.23%)	*p* = 0.149	86.75	8.86	88.48	43.33	99.09	82.42	92.50	*p* ^a^ < 0.001 *
	No (N = 10)	6.40	2.67	7	1	10	5.5	8.00		9 (90.00%)	1 (10.00%)		66.21	17.10	70.15	33.94	86.67	60.00	78.33	
Alternatives	Yes (N = 159)	9.03	1.11	9	6	10	8.0	10.00	*p* < 0.001 *	158 (99.37%)	1 (0.63%)	*p* = 0.149	89.95	6.08	91.21	69.70	99.09	86.36	94.24	*p* ^a^ < 0.001 *
	No (N = 95)	8.06	1.87	8	1	10	7.0	9.50		92 (96.84%)	3 (3.16%)		79.23	11.79	82.42	33.94	94.24	75.61	87.58	
Ongoing information during hysteroscopy	Yes (N = 239)	8.79	1.38	9	1	10	8.0	10.00	*p* < 0.001 *	236 (98.74%)	3 (1.26%)	*p* = 0.192	87.22	8.11	88.48	51.21	99.09	83.18	92.73	*p* ^a^ < 0.001 *
	No (N = 15)	6.08	1.61	6	3	9	5.0	7.00		14 (93.33%)	1 (6.67%)		63.75	16.18	69.70	33.94	83.94	49.39	75.15	
Forewarning of moments of increased discomfort during hysteroscopy	Yes (N = 235)	8.75	1.44	9	1	10	8.0	10.00	*p* = 0.001 *	232 (98.72%)	3 (1.28%)	*p* = 0.245	86.92	8.76	88.48	43.03	99.09	82.88	92.73	*p* ^a^ < 0.001 *
	No (N = 19)	7.35	1.93	8	3	10	6.0	9.00		18 (94.74%)	1 (5.26%)		73.44	17.20	75.45	33.94	94.24	69.70	85.15	
Interrupting hysteroscopy due to pain	Yes (N = 125)—A	8.67	1.41	9	1	10	8.0	10.00	*p* = 0.04 * C > B	124 (99.20%)	1 (0.80%)	*p* = 0.258	87.63	8.54	89.70	51.21	99.09	83.64	93.64	*p* ^b^ < 0.001 * A, C > B
	No (N = 20)—B	7.44	2.38	8	3	10	5.5	9.75		19 (9.05%)	1 (5.0%)		70.96	15.96	77.12	33.94	89.70	61.89	82.80	
	I did not express pain (N = 109)—C	8.83	1.36	9	5	10	8.0	10.00		107 (98.17%)	2 (1.83%)		86.64	8.47	88.18	43.33	98.48	82.42	92.12	

*p* ^a^—2 groups comparison: Mann-Whitney test; >2 groups comparison: Kruskal-Wallis test + post-hoc analysis (Dunn test), *p* ^b^—chi-squared or Fisher’s exact test, SD—standard deviation, Q1—lower quartile, Q3—upper quartile, and * statistically significant (*p* < 0.05).

**Table 5:** The correlations between selected variables and expectation fulfillment, as well as the willingness to undergo repeat procedures among women subjected to office hysteroscopy.

Parameter	Meeting Expectations		Likelihood of Re-Admission				Total Satisfaction Score [%]	
	Spearman’s Correlation Coefficient	*p*	OR	95%CI		*p* ^a^	Spearman’s Correlation Coefficient	*p*
Comprehension	r = 0.527	*p* < 0.001 *	1.672	1.023	2.733	0.04 *	0.548	*p* < 0.001 *
Sense od security	r = 0.481	*p* < 0.001 *	1.193	0.759	1.877	0.444	0.513	*p* < 0.001 *
Quality of information	r = 0.549	*p* < 0.001 *	1.124	0.737	1.716	0.587	0.555	*p* < 0.001 *
Intensity of pain	r = −0.21	*p* < 0.001 *	0.801	0.527	1.218	0.299	−0.267	*p* < 0.001 *
Quality of Communication during hysteroscopy	r = 0.591	*p* < 0.001 *	1.355	1.004	1.828	0.047 *	0.507	*p* < 0.001 *

*p* ^a^—univariate logistic regression, OR—odds ratio, 95%CI—95% confidence interval, and * statistically significant (*p* < 0.05).

**Table 6:** The comparison of the assessment of expectation fulfillment and the willingness to be readmitted to the evaluated facility based on the type of medical service received.

Parameter		Surgery Via Laparotomy or Laparoscopy Under General Anesthesia—A (N = 139)	Hysteroscopy Under General Anesthesia—B (N = 74)	Hysteroscopy Under Local Anesthesia—C (N = 254)	Hormonal Diagnostics of Menstrual Disorders or Infertility—D (N = 246)	Others—E (N = 77)	*p*
Meeting expectations	Mean (SD)	8.66 (1.63)	8.65 (1.49)	8.67 (1.51)	7.57 (1.78)	8.75 (1.73)	*p* < 0.001 * E, C, A, B > D
	Median (quartiles)	9 (8–10)	9 (8–10)	9 (8–10)	8 (7–9)	9 (8–10)	
	Range	0–10	4–10	1–10	1–10	1–10	
Likelihood of re-hospitalization	Yes	135 (97.12%)	73 (98.65%)	250 (98.43%)	240 (97.56%)	76 (98.70%)	*p* = 0.967
	No	2 (1.44%)	1 (1.35%)	4 (1.57%)	6 (2.44%)	1 (1.30%)	
	Unknown	2 (1.44%)	0 (0.00%)	0 (0.00%)	0 (0.00%)	0 (0.00%)	
Total satisfaction score [%]	Mean (SD)	88.44 (11.67)	85.77 (13.2)	85.94 (10.09)	84.66 (15.48)	72.03 (30.4)	*p* = 0.003 * A > C, E D > E
	Median (quartiles)	92.31 (83.27–96.73)	89.42 (80.77–95)	88.18 (82.42–92.42)	88.33 (77.08–96.67)	85 (56.92–95.77)	
	Range	41.92–100	37.69–100	33.94–99.09	35–100	9.62–100	

*p*—Qualitative variables: chi-squared or Fisher’s exact test. Quantitative variables: Kruskal-Wallis test + post-hoc analysis (Dunn test), SD—standard deviation, quartiles: Q1 (lower quartile)—Q3 (upper quartile), and * statistically significant (*p* < 0.05).

## Data Availability

The original contributions presented in this study are included in the article/Supplementary Material. Further inquiries can be directed to the corresponding author(s).

## Supplementary Materials

The following supporting information can be downloaded at: https://www.mdpi.com/article/10.3390/healthcare13243244/s1, Figure S1: The network of significant relationships between patient-dependent and provider-dependent factors concerning selected aspects of patient experience; Figure S2: Actual (solid line) and desired (dotted line) models of the organizational culture of the studied public healthcare organization; Table S1: Characteristics of the participants with their responses regarding qualification for the procedure, preoperative period, preparation and conduct of outpatient hysteroscopy, postoperative period, and pre-discharge issues; Table S2: The correlations of age, domicile, education, and profession with general and mental/emotional health status; Questionnaire S1: Anonymous satisfaction survey for received healthcare services.

## Abbreviations

The following abbreviations are used in this manuscript:

GDP	Gross domestic product
CFA	Confirmatory factor analysis
OH	Office hysteroscopy
OCAI	Organizational Culture Assessment Instrument
SD	Standard deviation
SRMR	Standardized Root Mean Residual
CFI	Comparative Fit Index
RMSEA	Root Mean Square Error of Approximation
OR	Odds ratio
