# Supplementary material for: Women’s Satisfaction with Gynecological Healthcare Services in a Public Tertiary Facility: A Questionnaire Study
Source: Healthcare (Basel). 2025 Dec 11;13(24):3244. doi: 10.3390/healthcare13243244 (PMC12732743; doi:10.3390/healthcare13243244)
Supplement: Supplementary file 1 [file healthcare-13-03244-s001.zip › Questionnaire S1.pdf]

ANONIMOWA ANKIETA SATYSFAKCJI Z UDZIELONEGO ŚWIADCZENIA ZDROWOTNEGO
(ANONYMOUS SATISFACTION SURVEY FOR RECEIVED HEALTHCARE SERVICE)
INSTRUKCJA WYPEŁNIANIA
(FILLING INSTRUCTIONS)

1. BEZPIECZEŃSTWO DANYCH OSOBOWYCH ORAZ DOBROWOLNOŚĆ UDZIAŁU (PERSONAL DATA SECURITY AND VOLUNTARY PARTICIPATION)

- Ankieta, którą ma Pani przed sobą jest całkowicie anonimowa. Na żadnym etapie nie zostanie Pani poproszona o podanie informacji, które pozwoliłyby na Pani identyfikację. Wszelkie podane dane są ponadto chronione zgodnie z Rozporządzeniem Parlamentu Europejskiego i Rady (UE) 2016/679 z dnia 27 kwietnia 2016 r. w sprawie ochrony osób fizycznych w związku z przetwarzaniem danych osobowych i w sprawie swobodnego przepływu takich danych oraz uchylenia dyrektywy 95/46/WE (ogólnym rozporządzeniem o ochronie danych) (The survey you have before you is entirely anonymous. At no point will you be asked to provide information that would allow for your identification. All data provided is also protected in accordance with the Regulation (EU) 2016/679 of the European Parliament and Council of April 27, 2016, concerning the protection of individuals with regard to the processing of personal data and the free movement of such data, and repealing Directive 95/46/EC (General Data Protection Regulation).
- Ankieta umożliwi ocenę stopnia zadowolenia pacjenta z udzielonego świadczenia zdrowotnego i przyczyni się do udoskonalenia opieki i poprawy jakości świadczeń w podmiocie, w którym dobro pacjenta jest nadrzędną wartością (The survey will assess the degree of patient satisfaction with the healthcare services received and will contribute to the enhancement of care and improvement of service quality in the institution, where the well-being of the patient is of paramount importance).
- Udział w niniejszym badaniu jest całkowicie dobrowolny. Może Pani zrezygnować na każdym etapie. Nie będzie miało to wpływu na dalszy przebieg Pani hospitalizacji oraz zakres udzielonych świadczeń (Participation in this study is entirely voluntary. You may withdraw at any stage without it affecting the course of your hospitalization or the extent of services provided).
- Po wypełnieniu ankiety prosimy o jej zwrot w wyznaczone miejsce (After completing the survey, please return it to the designated location).

2. INFORMACJE DOTYCZĄCE TREŚCI ANKIETY I SPOSOBU ODPOWIADANIA

Pytania zawarte w ankiecie dotyczą udzielonego świadczenia zdrowotnego, będącego celem hospitalizacji w tutejszym podmiocie leczniczym. Staranne wypełnienie niniejszego formularza podniesie jakość zebranych informacji, co pozwoli na dokładniejsze opracowanie danych, podnosząc wartość uzyskanych wyników. Z tego względu zwracamy się z prośbą o zachowanie kilku zasad w trakcie jego wypełniania (The questions included in the survey pertain to the healthcare services received, which are the purpose of your hospitalization at this medical facility. Careful completion of this form will enhance the quality of the collected information, enabling more accurate data analysis and increasing the value of the obtained results. Therefore, we kindly request that you adhere to the following guidelines while completing it):

- na pytania proszę odpowiadać szczerze, w kwestionariuszu nie ma poprawnych i złych odpowiedzi (please answer the questions honestly; there are no correct or incorrect answers in the questionnaire),
- w białym polu obok najbardziej pasującej odpowiedzi prosimy zaznaczyć znak X (in the white box next to the most appropriate answer, please indicate your response with an "X."),
- zazwyczaj najbardziej prawdziwe jest pierwsze odczucie, więc proszę nie zastanawiać się zbyt długo nad pytaniami (generally, your first impression is the most accurate, so please do not spend too long contemplating the questions.),
- jeśli jednak uzna Pani, że chciałaby zmienić swoją odpowiedź, niezgodny z odczuciami znak X proszę otoczyć kółkiem i zaznaczyć bardziej pasującą wersję odpowiedzi stawiając znak X (however, if you believe you would like to change your answer, please circle the "X" that does not reflect your feelings and mark the more appropriate version of the answer with an "X").

CZ. I. INFORMACJE OGÓLNE:

1. **Wiek** (Age):
   <20 lat (<20 years)
   21-30 lat (21-30 years)
   31-40 lat (31-40 years)
   41-50 lat (41-50 years)
   >50 lat (>50 years)

2. **Cel hospitalizacji** (Purpose of hospitalization):
   Zabieg operacyjny drogą laparotomii lub laparoskopii w znieczuleniu ogólnym (Surgery via laparotomy or laparoscopy under general anesthesia)
   Zabieg histeroskopii w znieczuleniu ogólnym (Hysteroscopy under general anesthesia)
   Zabieg histeroskopii w znieczuleniu miejscowym (Hysteroscopy under local anesthesia)
   Diagnostyka hormonalna zaburzeń miesiączkowania lub niepłodności (Hormonal diagnostics of menstrual disorders or infertility)
   Inne (other):
   ……………………………………………………………………………………………….

3. **Proszę wskazać miejsce zamieszkania** (Please indicate your domicile):

   wieś (village)
   miasto poniżej 50 000 mieszkańców (a city with less than 50,000 inhabitants)
   miasto 50 000 - 100 000 mieszkańców (city with 50,000 - 100,000 inhabitants)
   miasto powyżej 100 000 mieszkańców (a city with over 100,000 inhabitants)
   miasto powyżej 1000 000 mieszkańców (a city with over 1,000,000 inhabitants)

4. **Jaki poziom wykształcenia Pani posiada?** (What is your level of education?)
   Podstawowy (Elementary)
   Średni (Middle)
   Zawodowy (Technical)
   Wyższy (Higher)

5. **Czy wykonywany przez Panią zawód wymaga wiedzy medycznej?** (Does your profession require medical knowledge?)
   TAK (Yes)
   NIE (No)

6. **Jak ogólnie ocenia Pani stan swojego ogólnego zdrowia?** (How do you assess your general health?)
   doskonały (excellent)
   bardzo dobry (very good)
   dobry (good)
   średni (average)
   zły (poor)

7. **Jak ogólnie ocenia Pani stan swojego zdrowia psychicznego lub emocjonalnego?** (How would you rate your overall mental or emotional health?)
   doskonały excellent)
   bardzo dobry (very good)
   dobry (good)
   średni (average)
   zły (poor)

8. **Czy obecnie leczy się Pani lub leczyła w przeszłości z powodu depresji, zaburzeń nastroju?** (Are you currently being treated or have you been treated in the past for depression or mood disorders?)
   TAK (Yes)
   NIE (No)

9. **Czy pozostaje Pani w związku czy żyje Pani sama?** (Are you in a relationship or do you live alone?)
   w związku (in a relationship)
   sama (single)

10. **Jak długi był Pani czas oczekiwania na termin przyjęcia od momentu zgłoszenia się ze skierowaniem do placówki?** (How long was your waiting time for an admission date from the moment you reported your referral to the facility?)
   < 2 tygodnie (< 2 weeks)
   2 tygodnie - 1 miesiąc (2 weeks - 1 month)
   1-3 miesiące (1 – 3 months)
   3-6 miesiący (3-6 months)
   >6 miesięcy (>6 months)

CZ. II. PYTANIA DOTYCZĄCE BADANIA KWALIFIKACYJNEGO DO ZABIEGU (DOTYCZY TYLKO LECZENIA OPERACYJNEGO)

11. **Proszę wskazać jakie jest u Pani wskazanie do leczenia operacyjnego** (Please specify what the indication for surgical treatment is in your case)
   nowotwór złośliwy (malignancy)
   mięśniak macicy (myoma)
   polip trzonu macicy (uterine polyp)
   torbiel/guz jajnika (ovarian tumor/cyst)
   niepłodność (infertility)
   wypadanie narządu rodnego (pelvic organ prolapse)
   nietrzymanie moczu (urinary incontinence)
   nieprawidłowe krwawienia z dróg rodnych (abnormal uterine bleeding)
   powikłania poprzedniego zabiegu operacyjnego, w tym cięcia cesarskiego (complications of previous surgery, including cesarean section)
   ciąża pozamaciczna (extrauterine pregnancy)

12. **Czy w trakcie badania ginekologicznego został Pani zapewniony odpowiedni komfort?** (Were you provided with adequate comfort during the gynecological examination?)
   TAK (Yes)

NIE (No)

13. **Czy miała Pani możliwość zadawania pytań?** (Did you have the opportunity to ask any questions?)
    TAK (Yes)
    NIE (No) → proszę przejść do pytania nr 15 (go to the question nr 15)

14. **Czy uzyskała Pani odpowiedź na wszelkie nurtujące Panią pytania?** (Did you receive answers to all your questions?)
    TAK (Yes)
    NIE (No)

15. **Czy na rozmowę dotyczącą planowanego zabiegu lekarz przeznaczył wystarczająco dużo czasu?** (Did the doctor allocate enough time to discuss the planned procedure?)
    TAK (Yes)
    NIE (No)

16. **W skali od 0 do 10, gdzie 0 oznacza całkowity brak zrozumienia, a 10 to rozwianie wszelkich wątpliwości, jak bardzo klarownie zostały przekazane Pani informacje odnośnie planowanego zabiegu?** (On a scale from 0 to 10, where 0 indicates a complete lack of understanding and 10 indicates all doubts have been resolved, how clearly was the information regarding the planned procedure conveyed to you?)
    0
    1
    2
    3
    4
    5
    6
    7
    8
    9
    10

17. **Czy podczas badania przed planowym zabiegiem lekarz przedstawił alternatywne sposoby leczenia i zapytał, jaki sposób leczenia uważa Pani za najlepszy dla siebie?** (During the examination prior to the planned procedure, did the doctor present alternative treatment options and ask which treatment method you consider to be the best for yourself?)
    TAK (Yes)
    NIE (No)

18. **Czy dzięki uzyskanym informacjom czuła się Pani bezpieczniej/odczuwała Pani mniejszy niepokój decydując się na zabieg?** (Did the information you received make you feel safer or experience less anxiety when deciding to undergo the procedure?)
    TAK (Yes)
    NIE (No)

19. **W skali od 0 do 10, gdzie 0 to całkowity brak poczucia bezpieczeństwa, a 10 całkowity brak niepokoju, jak bardzo bezpiecznie czuła się Pani decydując na zabieg?** (On a scale from 0 to 10, where 0 indicates a complete lack of a sense of security and 10 indicates a complete absence of anxiety, how safe did you feel when deciding to undergo the procedure?)
    - 0
    - 1
    - 2
    - 3
    - 4
    - 5
    - 6
    - 7
    - 8
    - 9
    - 10

20. **Czy przed przyjęciem do szpitala została Pani poinstruowana o konieczności wykonania badań dodatkowych (np. badań laboratoryjnych z krwi, bakteriologicznych wymazów z dróg rodnych) oraz czy została Pani w sposób prosty i zrozumiały poinstruowana jak poprawnie przygotować się do zabiegu?** (Before your hospital admission, were you informed about the need for additional tests, e.g., blood tests, bacterial swabs, and given clear instructions on how to prepare for the procedure?)
    - TAK (Yes)
    - NIE (No)

21. **W skali od 0 do 10, jak ocenia Pani jakość przekazania informacji na temat zaplanowanego zabiegu operacyjnego?** (On a scale from 0 to 10, how would you rate the quality of the information provided about the planned surgical procedure?)
    - 0
    - 1
    - 2
    - 3
    - 4
    - 5
    - 6
    - 7
    - 8
    - 9
    - 10

22. **Czy oczekiwała Pani jakichkolwiek informacji, które nie zostały udzielone w trakcie konsultacji przed zabiegiem? Proszę wskazać jakich?** (Did you expect any information that was not provided during the pre-procedure consultation? If yes, please specify.
    - TAK (Yes)
        - Jeśli tak (If so):
          ……………………………………………………………………
          ……………………………………………………………………
          ……………………………………………………………………
          ……………………………………………………………………

……………………………………………………………………
……………………………………………………………………
……………………………………………………………………
……………………………………………………………………
……

NIE (No)

CZ. III. PYTANIA DOTYCZĄCE OKRESU PRZEDOPERACYJNEGO (DOTYCZY TYLKO LECZENIA OPERACYJNEGO)

23. **Czy miejsce oczekiwania na zabieg zapewniało Pani odpowiedni komfort?** (Did the waiting area for the procedure provide you with adequate comfort?)
    - TAK (Yes)
    - NIE (No)

24. **Czy w trakcie badań dodatkowych (pobranie krwi, EKG, itp.) został Pani zapewniony odpowiedni komfort i warunki?** (During the additional tests, e.g., blood draw, ECG, were you provided with adequate comfort?)
    - TAK (Yes)
    - NIE (No)

25. **Czy została Pani w sposób prosty i zrozumiały poinstruowana jak będzie przebiegało przygotowanie do zabiegu?** (Were you given clear and understandable instructions on how to prepare for the procedure?)
    - TAK (Yes)
    - NIE (No)

26. **W skali od 0 do 10, gdzie 0 oznacza całkowity brak zrozumienia, a 10 to rozwianie wszelkich wątpliwości, jak bardzo klarownie podczas tej konsultacji zostały przekazane Pani informacje odnośnie planowanego zabiegu**? (On a scale from 0 to 10, where 0 means no understanding at all and 10 means all doubts resolved, how clearly were the details about the planned procedure communicated to you during this consultation?)
    - 0
    - 1
    - 2
    - 3
    - 4
    - 5
    - 6
    - 7
    - 8
    - 9
    - 10

27. **Czy zostały Pani jasno i klarownie przekazane informacje dotyczące sposobu znieczulenia w trakcie zabiegu**? (Were you provided with clear and understandable information regarding the method of anesthesia during the procedure?)
    - TAK (Yes)

NIE (No)

CZ. IV. W TRAKCIE ZABIEGU (DOTYCZY TYLKO HISTEROSKOPII W ZNIECZULENIU MIEJSCOWYM)

28. **Czy w okresie poprzedzającym zabieg histeroskopii zostały zalecone leki do stosowania miejscowego (np. żel, krem dopochwowy)?** (Were any local medications, e.g., gel, vaginal cream, recommended for use prior to the hysteroscopy?)
    TAK (Yes); proszę wybrać właściwe (choose the correct one): Oekolp, Mucovagin
    NIE (No)

29. **Czy w trakcie zabiegu była Pani informowana o czynnościach, które operator zamierza wykonać przed ich faktycznym rozpoczęciem?** (Were you informed about the operator's planned actions before they actually started during the procedure?)
    TAK (Yes) → proszę ominąć pytanie nr 31 (skip the question no 31)
    NIE (No) → proszę przejść do pytania 31 (go to the question nr 31)

30. **Czy dzięki uzyskiwanym informacjom czuła się Pani bezpieczniej i bardziej komfortowo?** (Did the information you received make you feel safer and more comfortable?)
    TAK (Yes)
    NIE (No)

31. **Czy przez brak dostatecznych bieżących informacji dotyczących przebiegu zabiegu odczuwała Pani większy niepokój i dyskomfort**? (Did the lack of sufficient ongoing information about the procedure make you feel more anxious and uncomfortable?)
    TAK (Yes)
    NIE (No)

32. **W skali od 0 do 10, gdzie 0 to całkowity brak komfortu i poczucia bezpieczeństwa, a 10 maksymalny oczekiwany poziom komfortu i całkowite poczucie bezpieczeństwa/brak niepokoju, jak czuła się Pani podczas histeroskopii?** (On a scale from 0 to 10, where 0 indicates complete discomfort and lack of security, and 10 indicates maximum expected comfort and total sense of safety/no anxiety, how did you feel during hysteroscopy?)
    0
    1
    2
    3
    4
    5
    6
    7
    8
    9
    10

33. **Czy operator w trakcie wykonywanych czynności uprzedzał o momentach z możliwymi silniejszymi odczuciami bólu i możliwym większym dyskomfortem?** (Did the operator warn you about moments that might involve stronger pain or increased discomfort during the procedure?)

    TAK (Yes)
    NIE (No)

34. **Czy na Pani komunikat o odczuwaniu bólu operator przerywał wykonywane czynności? (**Did the operator pause their actions in response to your communication about experiencing pain?)

    TAK (Yes)
    NIE (No)
    NIE ZGŁASZAŁAM BÓLU (I did not express pain)

35. **W skali od 0 do 10, gdzie 0 to totalny brak dolegliwości bólowych, a 10 najgorszy ból dotąd odczuwany w życiu/ból nie do zniesienia, jak bardzo bolesny był dla Pani przeprowadzony zabieg histeroskopii? (**On a scale from 0 to 10, where 0 indicates no pain at all and 10 represents the worst pain you've ever experienced/ unbearable pain, how painful was the hysteroscopy procedure for you?)

    0
    1
    2
    3
    4
    5
    6
    7
    8
    9
    10

36. **W skali od 0 do 10, gdzie 0 to całkowity brak komunikacji i zrozumienia, a 10 komunikacja na najwyższym wyobrażalnym dla Pani poziomie, jak ocenia Pani komunikację między Panią a operatorem w trakcie zabiegu?** (On a scale from 0 to 10, where 0 is no communication and 10 is the highest possible level, how would you rate the communication between you and the operator during the procedure?)

    0
    1
    2
    3
    4
    5
    6
    7
    8
    9
    10

37. Czy w przyszłości zdecydowałaby się Pani poddać histeroskopii w znieczuleniu miejscowym, gdyby zaistniała taka potrzeba? (Would you consider undergoing hysteroscopy with local anesthesia in the future if necessary?)

TAK (Yes)
NIE (No)

CZ. V. PO ZABIEGU (DOTYCZY TYLKO LECZENIA OPERACYJNEGO)

38. Jak długi był czas oczekiwania od momentu przyjęcia do oddziału do momentu wykonania zabiegu? (What was the waiting time from admission to the department until the surgery was performed?)

zabieg został wykonany tego samego dnia (the procedure was performed on the same day)
1 dzień (the procedure was performed the next day)
2 dni (the procedure was performed 2 days after my admission)
więcej niż 2 dni (more than 2 days)

39. Czy w trakcie badania ginekologicznego po zabiegu otrzymała Pani informacje dotyczące procesu rekonwalescencji, spodziewanych i możliwych następstw oraz efektów? (During the gynecological examination after the surgery, did you receive information about the recovery process, expected outcomes, and potential effects?)

TAK (Yes)
NIE (No)

40. Czy została Pani poinformowana o mogących wystąpić niepokojących objawach wymagających pilnej interwencji lekarskiej? (Were you informed about any concerning symptoms that may require urgent medical intervention?)

TAK (Yes)
NIE (No)

41. Czy po zabiegu, przed wypisem była Pani badana przez lekarza wykonującego zabieg operacyjny? (After the procedure, before discharge, were you examined by the physician who performed the surgery?)

TAK (Yes)
NIE (No)

42. Czy po zabiegu, przed wypisem rozmawiała Pani z lekarzem wykonującym zabieg operacyjny? (After the procedure, before discharge, did you speak with the physician who performed the surgery?)

TAK (Yes)
NIE (No)

CZ. VI. PRZED WYPISEM ZE SZPITALA

43. Czy w trakcie hospitalizacji personel był pomocny w takim stopniu, w jakim Pani oczekiwała? (During your hospitalization, was the staff as helpful as you expected?)

Zdecydowanie tak (Definitely yes)

Raczej tak (Rather yes)
Nie (No)

44. **Czy w trakcie hospitalizacji personel traktował Panią uprzejmie i z szacunkiem?** (During your hospitalization, did the staff treat you politely and with respect?)
    Zdecydowanie tak (Definitely yes)
    Raczej tak (Rather yes)
    Nie (No)

45. **Czy w dniu wypisu miała Pani możliwość zadawania pytań?** (On the day of your discharge, did you have the opportunity to ask questions?)
    TAK (Yes)
    NIE (No) → proszę przejść do pytania nr 48 (go to the question nr 48)

46. **Czy lekarz zachęcał Panią do zadawania pytań?** (Did the doctor encourage you to ask questions?)
    TAK (Yes)
    NIE (No)

47. **Czy uzyskała Pani odpowiedź na wszelkie nurtujące Panią pytania**? (Did you receive answers to all the questions that concerned you?)
    TAK (Yes)
    NIE (No)

48. **Czy lekarz przeznaczył Pani wystarczająco dużo czasu?** (Did the doctor devote enough time with you during the discharge consultation?)
    TAK (Yes)
    NIE (No)

49. **W skali od 0 do 10, gdzie 0 oznacza całkowity brak spełnionych oczekiwań, a 10 hospitalizację w zgodzie z najwyższymi wyobrażalnym dla Panie standardami, jak oceniłaby Pani całą hospitalizację?** (On a scale from 0 to 10, where 0 means no expectations were met and 10 means hospitalization met your highest possible standards, how would you rate your overall hospitalization?)
    0
    1
    2
    3
    4
    5
    6
    7
    8
    9
    10

50. **Czy zdecydowałaby się Pani na ponowną hospitalizację w tutejszym ośrodku, gdyby było to konieczne?** (Would you choose to be hospitalized again at this facility if necessary?)
    tak (yes)
    nie (no)

51. **Czy chciałaby Pani wskazać jakieś pozytywne aspekty hospitalizacji/zabiegu, które nie były dotąd poruszone w kwestionariuszu?** (Would you like to mention any positive aspects of your hospitalization/procedure that have not yet been addressed in the questionnaire?)

TAK (Yes)

- Jeśli tak (If yes):
  ………………………………………………………………………
  ………………………………………………………………………
  ………………………………………………………………………

NIE (No)

52. **Czy chciałaby Pani wskazać jakieś negatywne aspekty hospitalizacji/zabiegu, które nie były dotąd poruszone w kwestionariuszu? (**Would you like to highlight any negative aspects of your hospitalization/procedure that have not yet been addressed in the questionnaire?)

TAK (Yes)

- Jeśli tak (If yes):
  ………………………………………………………………………
  ………………………………………………………………………
  ………………………………………………………………………

NIE (No)
