# Supplementary material for: Women’s Satisfaction with Gynecological Healthcare Services in a Public Tertiary Facility: A Questionnaire Study
Source: Healthcare (Basel). 2025 Dec 11;13(24):3244. doi: 10.3390/healthcare13243244 (PMC12732743; doi:10.3390/healthcare13243244)
Supplement: Supplementary file 1 [file healthcare-13-03244-s001.zip › Supplementary Figure S2.pdf]

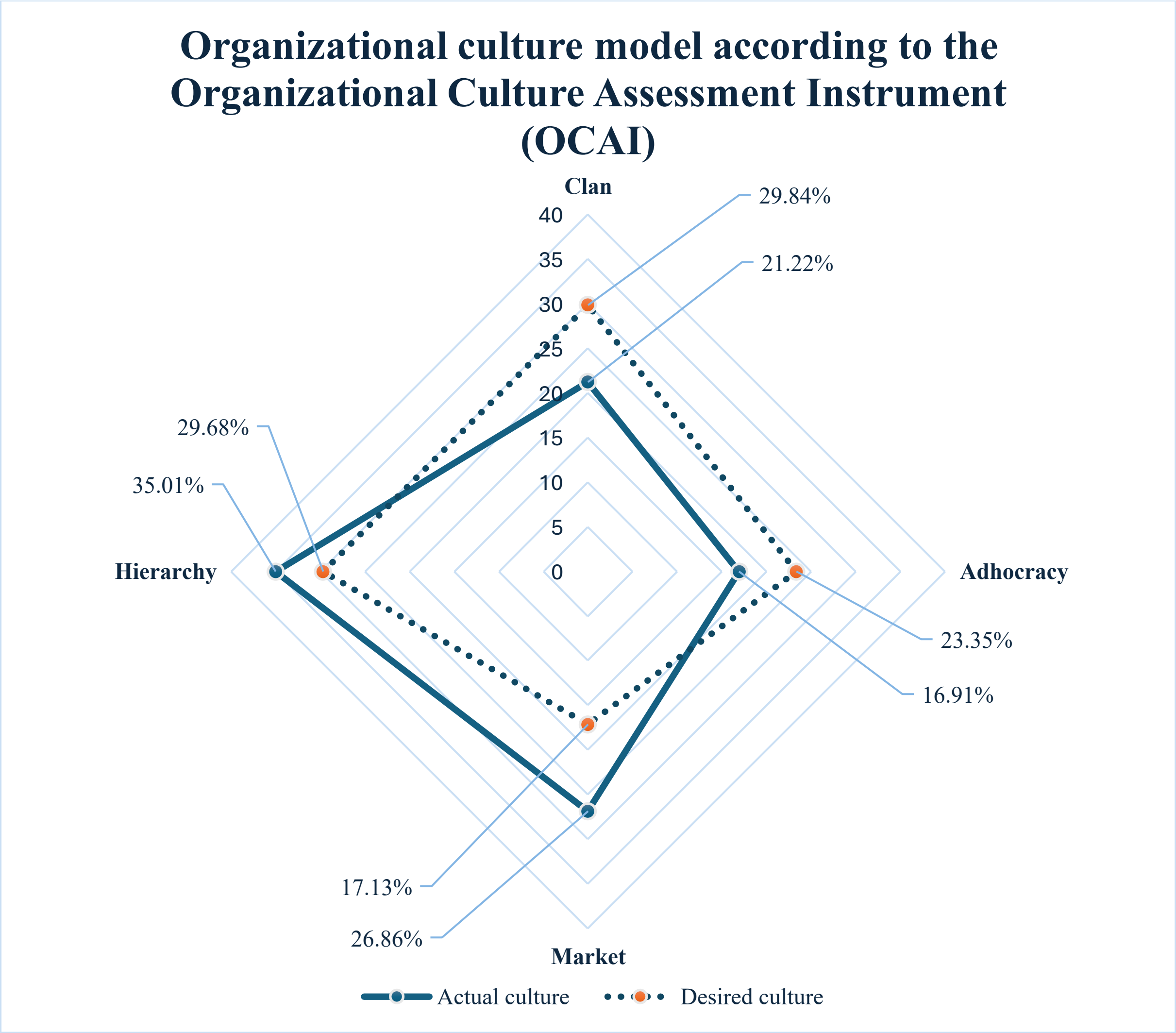

Organizational culture model according to the Organizational Culture Assessment Instrument (OCAI)
Clan
Adhocracy
Market
Hierarchy
40
35
30
25
20
15
10
5
0
29.84%
21.22%
29.68%
35.01%
23.35%
16.91%
17.13%
26.86%
Actual culture
Desired culture
