# Supplementary material for: Women’s Satisfaction with Gynecological Healthcare Services in a Public Tertiary Facility: A Questionnaire Study
Source: Healthcare (Basel). 2025 Dec 11;13(24):3244. doi: 10.3390/healthcare13243244 (PMC12732743; doi:10.3390/healthcare13243244)
Supplement: Supplementary file 1 [file healthcare-13-03244-s001.zip › Table S1.pdf]

Table S1. Characteristics of the participants with their responses regarding qualification for the procedure, preoperative period, preparation and conduct of outpatient hysteroscopy, postoperative period, and pre-discharge issues.

**Characteristics of the respondents with respect to demographic data, the purpose of hospitalization, and health issues**		
**Parameter**	**Value**	**Total (N=790)**
Age	< 20 years	47 (5.95%)
	21-30 years	241 (30.51%)
	31-40 years	274 (34.68%)
	41-50 years	121 (15.32%)
	>50 years	107 (13.54%)
Purpose of hospitalization	Surgery via laparotomy or laparoscopy under general anesthesia	139 (17.59%)
	Hysteroscopy under general anesthesia	74 (9.37%)
	Hysteroscopy under local anesthesia	254 (32.15%)
	Hormonal diagnostics of menstrual disorders or infertility	246 (31.14%)
	Others	77 (9.75%)
Domicile	Village	252 (31.90%)
	City < 50,000 inhabitants	96 (12.15%)
	City 50,000-100,000 inhabitants	68 (8.61%)
	City 100,000-1,000,000 inhabitants	220 (27.85%)
	City > 1,000,000 inhabitants	154 (19.49%)
Education	Elementary	26 (3.29%)
	Middle	193 (24.43%)
	Technical	44 (5.57%)
	Higher	527 (66.71%)
Health-related profession	Yes	145 (18.35%)
	No	645 (81.65%)
General health	Excellent	24 (3.04%)
	Very good	261 (33.04%)
	Good	402 (50.89%)
	Average	94 (11.90%)
	Poor	9 (1.14%)
Mental/Emotional Health	Excellent	56 (7.09%)
	Very good	275 (34.81%)
	Good	315 (39.87%)
	Average	129 (16.33%)
	Poor	15 (1.90%)
Mood disorders/ depression	Yes	133 (16.84%)
	No	657 (83.16%)
Relationship Status	Single	163 (20.63%)
	In a relationship	627 (79.37%)
Waiting time for the admission	< 2 weeks	184 (23.29%)
	2 weeks - 1 month	124 (15.70%)
	1-3 months	219 (27.72%)
	3-6 months	193 (24.43%)
	> 6 months	70 (8.86%)
Indication for surgical treatment	Malignancy	10 (1.27%)
	Uterine fibroid	70 (8.86%)
	Uterine polyp	158 (20.00%)
	Ovarian tumor/cyst	64 (8.10%)
	Infertility	124 (15.70%)
	Pelvic organs prolapse	22 (2.78%)
	Urinary incontinence	11 (1.39%)
	Abnormal uterine bleeding	38 (4.81%)
	Complications of previous surgery	17 (2.15%)
	Extrauterine pregnancy	29 (3.67%)
	Hormonal disorders	247 (31.27%)
**Responses provided by the respondents to inquiries regarding the qualification for the procedure and the preoperative period**		
**Parameter**	**Value**	**Total (N=526)**
Comfort during examination	Yes	510 (96.96%)
	No	16 (3.04%)
Asking questions	Yes	524 (99.62%)
	No	2 (0.38%)
Allotting enough time	Yes	497 (94.49%)
	No	29 (5.51%)

Comprehension	Mean (SD)	8.89 (1.51)
	Median (quartiles)	10 (8-10)
	Range	1-10
Alternatives	Yes	359 (68.25%)
	No	167 (31.75%)
Feeling safe	Yes	465 (88.4%)
	No	61 (11.6%)
Sense of security	Mean (SD)	8.08 (1.91)
	Median (quartiles)	8 (7-10)
	Range	0-10
Pre-admission instructions	Yes	468 (88.97%)
	No	58 (11.03%)
Quality of information	Mean (SD)	8.43 (2.12)
	Median (quartiles)	9 (8-10)
	Range	0-10
Expectation to provide missing information	Yes	53 (10.08%)
	No	473 (89.92%)
Comfort while waiting	Yes	467 (88.78%)
	No	59 (11.22%)
Comfort during additional tests	Yes	504 (95.82%)
	No	22 (4.18%)
Instruction on preparation for surgery	Yes	490 (93.16%)
	No	36 (6.84%)
Comprehension of the planned procedure	Mean (SD)	8.79 (1.64)
	Median (quartiles)	9 (8-10)
	Range	0-10
Information about anesthesia	Yes	473 (89.92%)
	No	53 (10.08%)
**Responses provided by the respondents to questions concerning the preparation for and conduct of outpatient hysteroscopy**		
**Parameter**	**Value**	**Total (N=254)**
Pre-hysteroscopy vaginal preparation	Yes	53 (20.87%)
	No	201 (79.13%)
Ongoing information during hysteroscopy	Yes	239 (94.09%)
	No	15 (5.91%)
Sense of comfort and safety during hysteroscopy	Mean (SD)	8.46 (1.89)
	Median (quartiles)	9 (8-10)
	Range	1-10
Forewarning of moments of increased discomfort during hysteroscopy	Yes	235 (92.52%)
	No	19 (7.48%)
Interrupting hysteroscopy due to pain	Yes	125 (49.21%)
	No	20 (7.87%)
	I did not express pain	109 (42.91%)
Intensity of pain during hysteroscopy	Mean (SD)	5.19 (2.5)
	Median (quartiles)	5 (3-7)
	Range	0-10
Quality of communication during hysteroscopy	Mean (SD)	8.9 (1.93)
	Median (quartiles)	10 (9-10)
	Range	1-10
Willingness to undergo hysteroscopy in the future	Yes	238 (93.70%)
	No	16 (6.3%)
**Responses provided by the respondents to questions regarding the course of the postoperative period**		
**Parameter**	**Value**	**Total (N=526)**
Waiting time for the surgery	The procedure was performed on the same day	289 (54.94%)
	The procedure was performed the next day	159 (30.23%)
	The procedure was performed 2 days after admission	36 (6.84%)
	More than 2 days	42 (7.98%)
Post-surgery information	Yes	470 (89.35%)
	No	56 (10.65%)
Information on possible postoperative complications	Yes	427 (81.18%)
	No	99 (18.82%)
Discharge examination by the operator	Yes	238 (45.25%)
	No	288 (54.75%)
Discharge consultation with the operator	Yes	341 (64.83%)
	No	185 (35.17%)
**Responses provided by respondents to inquiries regarding the period preceding discharge**		
**Parameter**	**Value**	**Total (N=790)**
Expected staff support	Definitely yes	555 (70.25%)
	Rather yes	227 (28.73%)
	No	8 (1.01%)
Treatment with politeness and respect	Definitely yes	644 (81.52%)

	Rather yes	142 (17.97%)
	No	4 (0.5%)
Opportunity to ask questions at discharge	Yes	762 (96.46%)
	No	28 (3.54%)
Enough time during discharge consultation	Yes	708 (89.62%)
	No	82 (10.38%)
Meeting expectations	Mean (SD)	8.33 (1.71)
	Median (quartiles)	9 (7-10)
	Range	0-10
Likelihood of re-hospitalization	Yes	774 (97.97%)
	No	16 (2.03%)
Negative comments	Yes	168 (21.27%)
	No	622 (78.73%)
Positive comments	Yes	157 (19.87%)
	No	633 (80.13%)
**Women's Satisfaction**		
**Parameter**	**Value**	**Total (N=790)**
Total satisfaction score [%]	Mean (SD)	84.61 (15.96)
	Median (quartiles)	88.48 (79.62, 95.77)
	Range	9.62-100

SD - standard deviation, quartiles: Q1 (lower quartile), Q3 (upper quartile), range: minimal value-maximal value
