# Supplementary material for: Women’s Satisfaction with Gynecological Healthcare Services in a Public Tertiary Facility: A Questionnaire Study
Source: Healthcare (Basel). 2025 Dec 11;13(24):3244. doi: 10.3390/healthcare13243244 (PMC12732743; doi:10.3390/healthcare13243244)
Supplement: Supplementary file 1 [file healthcare-13-03244-s001.zip › Table S2.pdf]

Table S2. The correlations of age, domicile, education, and profession with general and mental/emotional health status.

Parameter	Group	General health					p	Mental/Emotional Health					p
		Perfect	Very good	Good	Average	Poor		Perfect	Very good	Good	Average	Poor	
Age	< 20 years (N=47)	0 (0.00%)	15 (31.91%)	24 (51.06%)	7 (14.89%)	1 (2.13%)	p<0.001*	3 (6.38%)	13 (27.66%)	13 (27.66%)	15 (31.91%)	3 (6.38%)	p<0.001*
	21-30 years (N=241)	2 (0.83%)	98 (40.66%)	118 (48.96%)	20 (8.30%)	3 (1.24%)		12 (4.98%)	89 (36.93%)	81 (33.61%)	51 (21.16%)	8 (3.32%)	
	31-40 years (N=274)	13 (4.74%)	97 (35.40%)	138 (50.36%)	24 (8.76%)	2 (0.73%)		21 (7.66%)	91 (33.21%)	123 (44.89%)	35 (12.77%)	4 (1.46%)	
	41-50 years (N=121)	7 (5.79%)	38 (31.40%)	60 (49.59%)	15 (12.40%)	1 (0.83%)		15 (12.40%)	49 (40.50%)	46 (38.02%)	11 (9.09%)	0 (0.00%)	
	>50 years (N=107)	2 (1.87%)	13 (12.15%)	62 (57.94%)	28 (26.17%)	2 (1.87%)		5 (4.67%)	33 (30.84%)	52 (48.60%)	16 (14.95%)	1 (0.93%)	
Domicile	Village (N=252)	10 (3.97%)	79 (31.35%)	131 (51.98%)	29 (11.51%)	3 (1.19%)	p=0.246	19 (7.54%)	87 (34.52%)	105 (41.67%)	39 (15.48%)	2 (0.79%)	p=0.853
	City < 50,000 inhabitants (N=96)	2 (2.08%)	38 (39.58%)	46 (47.92%)	9 (9.38%)	1 (1.04%)		7 (7.29%)	36 (37.50%)	35 (36.46%)	15 (15.62%)	3 (3.12%)	
	City 50,000-100,000. inhabitants (N=68)	0 (0.00%)	17 (25.00%)	37 (54.41%)	13 (19.12%)	1 (1.47%)		3 (4.41%)	20 (29.41%)	29 (42.65%)	14 (20.59%)	2 (2.94%)	
	City 100,000-1,000,000 inhabitants (N=220)	11 (5.00%)	76 (34.55%)	101 (45.91%)	29 (13.18%)	3 (1.36%)		12 (5.45%)	79 (35.91%)	90 (40.91%)	33 (15.00%)	6 (2.73%)	
	City > 1,000,000 inhabitants (N=154)	1 (0.65%)	51 (33.12%)	87 (56.49%)	14 (9.09%)	1 (0.65%)		15 (9.74%)	53 (34.42%)	56 (36.36%)	27 (17.53%)	3 (1.95%)	
Education	Elementary (N=26)	0 (0.00%)	9 (34.62%)	11 (42.31%)	4 (15.38%)	2 (7.69%)	p=0.001*	1 (3.85%)	9 (34.62%)	6 (23.08%)	9 (34.62%)	1 (3.85%)	p=0.118
	Middle (N=193)	3 (1.55%)	52 (26.94%)	105 (54.40%)	32 (16.58%)	1 (0.52%)		13 (6.74%)	54 (27.98%)	90 (46.63%)	32 (16.58%)	4 (2.07%)	
	Technical (N=44)	0 (0.00%)	8 (18.18%)	28 (63.64%)	8 (18.18%)	0 (0.00%)		3 (6.82%)	16 (36.36%)	21 (47.73%)	3 (6.82%)	1 (2.27%)	
	Higher (N=527)	21 (3.98%)	192 (36.43%)	258 (48.96%)	50 (9.49%)	6 (1.14%)		39 (7.40%)	196 (37.19%)	198 (37.57%)	84 (15.94%)	10 (1.9%)	
Health-related profession	Yes (N=145)	6 (4.14%)	43 (29.66%)	69 (47.59%)	25 (17.24%)	2 (1.38%)	p=0.165	12 (8.28%)	38 (26.21%)	58 (40.00%)	33 (22.76%)	4 (2.76%)	p=0.04*
	No (N=645)	18 (2.79%)	218 (33.80%)	333 (51.63%)	69 (10.70%)	7 (1.09%)		44 (6.82%)	237 (36.74%)	258 (40%)	95 (14.73%)	11 (1.71%)	

p - chi-squared or Fisher's exact test, * statistically significant (p<0.05)
