# Supplementary figures and images for: Women’s Satisfaction with Gynecological Healthcare Services in a Public Tertiary Facility: A Questionnaire Study
Source: Healthcare (Basel). 2025 Dec 11;13(24):3244. doi: 10.3390/healthcare13243244 (PMC12732743; doi:10.3390/healthcare13243244)

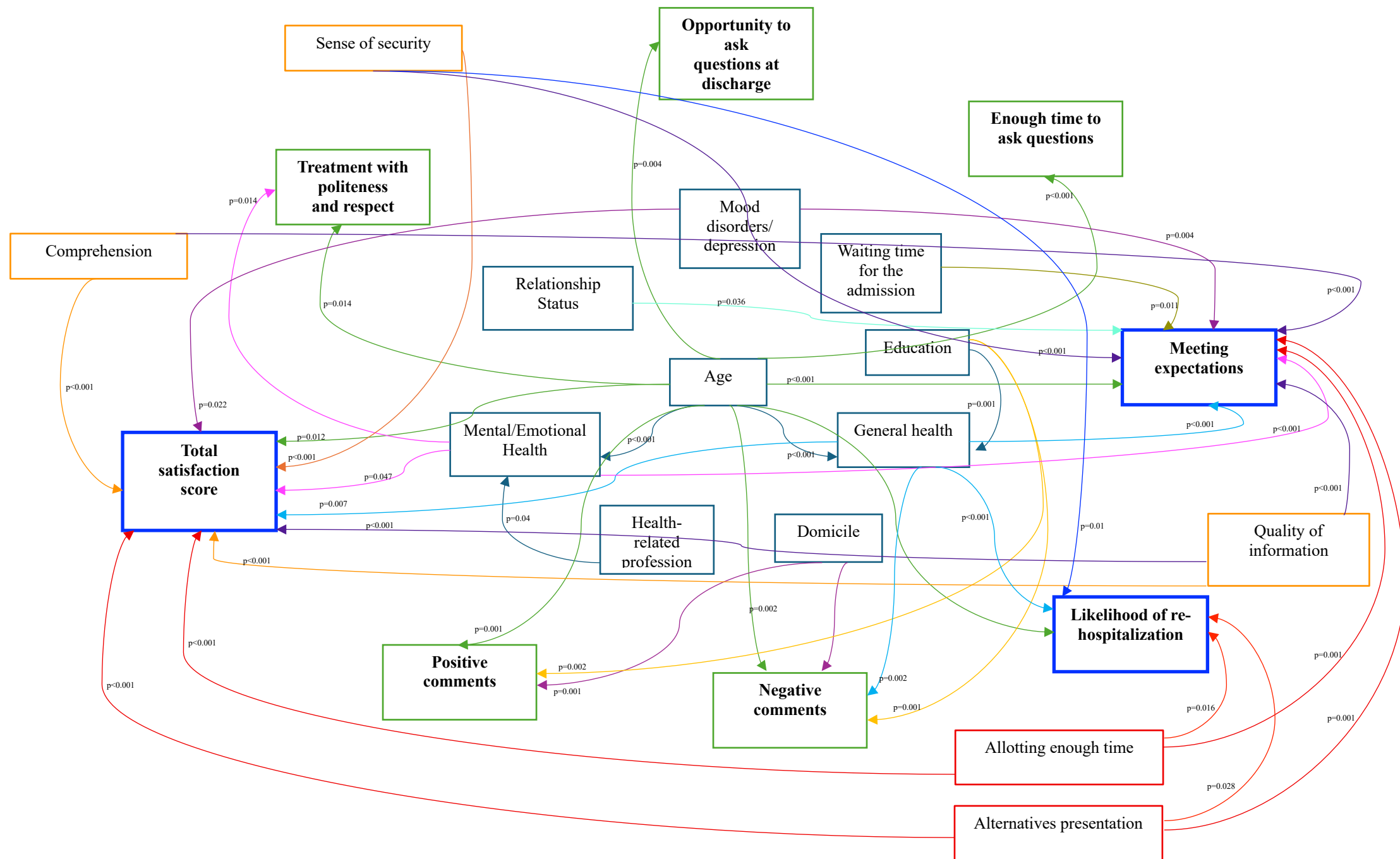

Supplement: Supplementary file 1 [file healthcare-13-03244-s001.zip › Supplementary Figure S1.pdf]
